# A comprehensive overview of a large-scale survey on inequality perceptions (IneqPer) in Italy

**DOI:** 10.3389/fsoc.2025.1620096

**Published:** 2025-07-18

**Authors:** Nevena Kulic, Olga Griaznova, Eleonora Clerici, Daniela Bellani, Debora Mantovani, Loris Vergolini, Francesco Scervini

**Affiliations:** ^1^Department of Political and Social Sciences, University of Pavia, Pavia, Italy; ^2^Department of Statistical Sciences, Catholic University of Sacro Cuore, Milan, Italy; ^3^Department of Political and Social Science, University of Bologna, Bologna, Italy

**Keywords:** inequality, perceptions, socio-economic status, gender, immigration, Italy, survey experiments

## Abstract

The article describes the content, the methodology and the selected results deriving from a large-scale cross-sectional IneqPer survey in Italy (*n* = 12,000, 2024/2025) aimed at understanding the determinants of inequality perceptions and their consequences for public opinion. The dataset offers novel dimensions that include the questions on global inequality and global redistribution, individual position in global distribution, the perceived social mobility in the society, perceived gender inequality and perceptions of discrimination against immigrants, among others. Moreover, for a number of questions, it offers a possibility of cross-validation with a range of recent datasets including European Social Survey (2020), European Value Study (2017), and the International Social Survey Program (2019). The first results show that Italians indeed recognize inequality along all dimensions (e.g., socio-economic inequality, gender inequality and inequality between migrants and natives), yet they do not strongly perceive themselves as personally affected by discrimination. Moreover, although cross-validation checks reveal a strong alignment between IneqPer data and other international value surveys, respondents in the IneqPer dataset tend to express slightly more progressive views.

## 1 Introduction

Inequality, defined as the unequal distribution of power, resources, and opportunities between different groups within a population, is one of the most critical challenges for world development. While extensive research has documented both current levels of inequality and their evolution over time (Mijs et al., 2022), significantly less attention has been paid to how people perceive these disparities. Yet, public perceptions of inequality are highly consequential in terms of social and economic behavior (Kelly and Enns, 2010), political consensus (Giugni and Grasso, 2019), political beliefs and participation (Knell and Stix, 2020), preferences for redistribution (Cruces et al., 2013), and social trust and cohesion (Han et al., 2021; Van Assche et al., 2023).

This article presents a novel dataset resulting from the project ‘Inequality between Reality and Perception (IneqPer)’, which investigates perceptions of inequality in Italy across three main dimensions: socio-economic status, gender, and immigration status. This topic is approached by integrating two dimensions of inequality: vertical inequalities, which pertain to differences in socio-economic status such as wealth and income, and horizontal inequalities, which arise between socially defined groups, for instance, between men and women, immigrants and natives (Ridgeway, 2019). This dual perspective allows us to capture not only disparities in individual resources, but also the structural and identity-based dimensions of inequality that shape people’s opportunities and outcomes (Stewart, 2001). Although societies vary significantly in the levels of income and wealth inequality (Neckerman and Torche, 2007), existing evidence has shown that people’s perceptions of these inequalities can differ markedly (Niehues, 2014). Moreover, people often have erroneous perceptions of their own position within the income distribution (Bellani et al., 2021). Perceptions of group-based inequalities are also critical for public support of policies aimed at social integration (Ridgeway, 2019), particularly regarding legal aspects (e.g., rights) and the economic status of these groups (e.g., labor market prospects).

The dataset on perceptions of inequality used in this study relies on the three overarching domains and incorporates metrics that measure subjective perceptions of the aforementioned inequalities, both of their general form and of their specific components. The dataset is, therefore, particularly timely, as it captures key challenges currently confronting Italy and other European countries in the areas of socio-economic and gender inequality, social integration, and migration. Given that public perceptions of inequality influence support for redistributive policies, social trust, and public attitudes toward marginalized groups (Cruces et al., 2013; Han et al., 2021), the survey contributes to a better understanding of public opinion in Italy, thereby informing more effective social and economic policies. The dataset adds to the growing body of national surveys with a focus on perceived inequalities, such as the Inequality Barometer in Germany (Bellani et al., 2021) and the Swiss Equality Barometer (Jans et al., 2024). However, while most existing datasets have focused on perceptions of socio-economic inequalities, the IneqPer project broadens this scope to include other dimensions of inequality.

The article is organized as follows. It first presents the Italian context, then introduces the survey, outlining its key components and the implementation process. Second, it presents an analysis of selected findings across the survey’s main dimensions. Finally, it assesses the generalizability of the key survey questions by comparing them with results from other established international surveys, focusing specifically on data collected in Italy.

## 2 An overview of the Italian context

What is Italy’s standing in terms of socio-economic inequality, gender-based disparities, and inequalities between migrants and the native population? Regarding socio-economic inequality, Italy is frequently cited as one of the most unequal European countries in terms of income distribution and social mobility (Schizzerotto and Marzadro, 2008), as corroborated by recent statistical data. In 2022, the top 10% of income earners accounted for 37.1% of total national income. While this figure is below the global average of 53.3%, it surpasses those of France, Germany, and the United Kingdom, where the corresponding shares were 34.0, 36.2, and 36.7%, respectively (The World Inequality Report, 2022). This pattern has shown little change over time: in 1998, for instance, the top decile held 34.8% of income, indicating only a modest increase over a 25-year period. Similarly, the share of income accruing to the bottom 50% of the population has declined only slightly, from 17.1% in 1998 to 16.6% in 2022—levels that remain consistently lower than those observed in France, Germany, and the United Kingdom at both time points. These trends are further supported by the Gini index which reflects comparatively higher inequality in Italy (34.8 in 2021 compared to 31.5 in France, 32.4 in Germany and 32.4 in the United Kingdom– World Bank, 2025^1^). Furthermore, the disproportionate tax burden on income as opposed to wealth has been identified as a structural factor inhibiting upward social mobility (Acciari et al., 2024).

As for gender inequality, Italy has been classified as a country with moderate to high levels, although it has shown progressive improvement over time. According to the European Institute for Gender Equality and its latest index for 2024 (based on 2022 data), Italy scored 69.2 out of 100 on the Gender Equality Index (GEI), slightly below the EU average of 71 (EIGE, 2024), and ranked 14th among the 27 EU member states. The index comprises six core dimensions: health, work, money, time, power, and knowledge. When the components of the index are considered separately, Italy performs relatively well in the domains of health (89.3) and money (80.6)—the latter encompassing net income, earnings, and risk of poverty. Conversely, in the dimensions related to employment, such as labor market participation and career advancement, the mean values for Italy are 65.5—below the EU average— as are measures of political and economic power (66.5). Nonetheless, Italy’s position on the index has improved steadily over time. Starting at a score of 53.3 in 2013, when the index was first introduced, it increased to 63.8 in 2019, 65.0 in 2020, and reached 68.2 in both 2021 and 2022. These developments align with broader trends of convergence among EU member states and a global shift toward greater gender equality (EIGE, 2024).

As for the third dimension of inequality, concerning disparities between migrants and natives, Italy is a relatively recent country of immigration, with the number of immigrants increasing significantly over the past three decades (Colucci, 2020). Despite this demographic shift, immigrants in Italy continue to experience poor labor market outcomes and a pronounced devaluation of their educational qualifications (Reyneri and Fullin, 2011). This is particularly evident in the wage gap between immigrants and natives: according to the most recent available data from 2015, Italy ranked among the top three EU countries with the largest pay disparities based on mean hourly wages, 29.6% overall, with a gap of 31.1% for men and 27.0% for women (Amo-Agyei, 2020, pp. 71, 75). Moreover, Italy lags behind many European counterparts in terms of comprehensive integration policies. The first immigration law to introduce integration-related measures (Law No. 40/1998) focused primarily on health and education and more specifically on recognition of rights for legally residing foreigners to access employment, education, and healthcare (Caneva, 2014). However, no substantial labor market integration policies have been implemented since. On the contrary, subsequent legislation—most notably Law No. 189/2002 (commonly referred to as the ‘Bossi-Fini Law’)—has further restricted immigration flows, producing adverse effects on immigrants’ integration into the workforce (Caneva, 2014).

## 3 Methodology

### 3.1 Survey design and sampling

Italy does not have large-scale, comprehensive data on perceptions of inequality. The most recent national surveys that have partially included perception-related questions are the ‘Aspetti della vita quotidiana’ (MULTISCOPO) surveys conducted by the Italian National Institute of Statistics (ISTAT). However, these surveys limit their questions to perceptions of individual health and economic circumstances rather than perceptions of inequality itself. Consequently, neither ISTAT nor any research groups have, to date, undertaken a systematic effort to collect data on perceptions of inequality in Italy. The International Social Survey Program (ISSP) includes a module on social inequality, which is repeated every 10 years, most recently in 2019. The questions, however, are limited to socio-economic inequality, with only occasional reference to other forms of inequality such as along ethnic, religious or gender lines. Given these limitations, the goal of the IneqPer survey is to address these gaps in data availability for Italy.

The IneqPer survey focuses exclusively on perceptions of inequality in areas such as national and global income inequality, static inequality and changes over time, gender inequality, and inequality between migrants and natives. It also includes questions on perceptions of both inequality and discrimination. It extends the work of ISSP and other international surveys by including in its design survey experiments. The dataset’s dual contribution thus lies in its provision of a general (and descriptive) survey on perceptions in Italy and its reliance on survey experiments to explore causal relationships. Table 1 presents a summary of the topics covered in the survey, while the original questionnaire is available as Supplementary material.

**Table 1:** Summary of the dimensions in the general part of the survey (*N* = 12,000) and the subject-specific modules (*N* = 2,000 each).

Topic	Variables
Socio-demographic characteristics	Age Gender Citizenship Migration background Type of settlement Education Marital status Children Fertility intentions Household composition Employment status Occupation Income Economic conditions Housing conditions Religion Media and sources of information Parental background
Attitudes	Politics Trust in institutions Discrimination Norms
Gender inequality	Perceived gender inequality Perceived trends of gender inequality Perceived gender inequality across regions Attitudes toward affirmative action policies
Income inequality	Perceived current type of inequality in Italy Desirable type of inequality in Italy Perceived past trends of inequality Expected future trends of inequality Perceived income inequality across regions Preferences for redistribution Perceived social mobility Determinants of success Perceived position in income distribution
Migration and ethnic inequality	Perceived predominant regions of origin of immigrants Perceived characteristics of immigrants and natives Perceived reasons for immigration Areas of ethnic discrimination Perceived trends of international migration
Global inequality	Perceived income inequality across countries Preferences for redistribution at the global level Perceived income inequality compared to the European Union Perceived position in income distribution at the global level
Subject-specific modules	
Income and social status	Perceived threshold of the poverty line Perceived position in income distribution with reference to the poverty line Preferences for redistribution
Gender-Based violence	Previous experience of violence Previous experience of violence in the workplace Opinions on gender-based violence in Italy Perceptions of normalization of violence
Climate change	Climate change literacy Worries about climate change Causes of climate change Support for climate change policies
Migration	Perceived ethnicity of immigrants Perceived share of immigrants Perceived share of children born in families where one or both parents are immigrants Perceived change in the share of children born in families where one or both parents are immigrants Attitudes toward immigrants

The IneqPer survey is an online, cross-sectional study employing Computer Assisted Web Interviewing (CAWI) that targeted the Italian population aged 18–70. The total sample comprised 12,000 participants. The survey was designed to be representative of the population along five key socio-demographic variables. To achieve this, quota sampling was employed to ensure that the sample reflects the demographic distribution across these dimensions. The quotas were defined by age group (18–24, 25–34, 35–44, 45–54, 55–64, and 65–70), gender (women, men, and other), macro-region in Italy (North-East, North-West, Centre, and South and Islands), education level [high (university or above), medium (high school), and low (elementary school)], and employment status (employed, unemployed, and inactive). The values were determined using the most recent Labor Force Surveys, including the 2020 edition for education and the 2023 edition for employment, and Eurostat 2021 for other variables.

These quotas were strictly followed during data collection, although some inconsistencies emerged between the intended and actual distributions. While the gender, age, and regional quotas were largely met, some discrepancies were noted for education and employment status. Specifically, individuals with low education were slightly underrepresented, while those with medium and high education were slightly overrepresented. Similarly, unemployed individuals were slightly overrepresented, whereas employed and inactive individuals were slightly underrepresented. To address these imbalances, post-stratification weights were created to adjust the sample and align it with the actual population distribution in Italy. These weights help mitigate potential biases and ensure that the results accurately reflect the broader demographic composition of Italy. Intended and actual quotas are presented in Table 2.^2^

**Table 2:** Intended versus actual quotas (*N* = 12,000).

Key socio-economic variables	Intended %	Actual %
Gender		
Male	49.70%	48.71%
Female	50.30%	51.08%
Other	Open	0.21%
Age		
18–24	10.20%	10.20%
25–34	15.60%	15.86%
35–44	18.70%	18.87%
45–54	23.70%	23.98%
55–64	21.40%	21.65%
65–70	10.40%	9.43%
Area		
North-West	26.60%	27.27%
North-East	19.50%	18.66%
Centre	19.80%	19.85%
South and Islands	34.10%	34.23%
Education		
Low	44.10%	33.46%
Medium	35.80%	42.84%
High	20.10%	23.70%
Labor market participation		
Employed	61.50%	57.52%
Unemployed	5.20%	10.86%
Not in the labor force	33.30%	31.62%

The first part of the IneqPer survey consisted of 62 general questions exploring perceptions of socio-economic status, gender, and immigration status. These questions were administered to the entire sample of 12,000 respondents. In addition to measuring perceptions of inequality and discrimination, the survey collected extensive socio-demographic data, including age, gender, marital status, household composition, fertility intentions, education level, and employment status, as well as respondents’ political attitudes and beliefs, religion, and preferences for redistribution.

The questionnaire incorporated both newly developed questions tailored specifically for the survey and established questions drawn from previous cross-national surveys to enable comparisons and cross-validation. For instance, survey questions addressing inequality in society (‘the type of society question’), in which respondents select images that best represent the structure of their society in terms of inequality, have been used since 1987 in the ISSP. Questions on the perceived drivers of success in society have also appeared in some ISSP waves. The question on the degree of current inequalities between men and women were asked in the 2012 Eurobarometer. However, because some of these questions have not been consistently asked over time or were only used in single waves and the most recent data are not available, these questions have been incorporated also in the IneqPer survey. The partial reliance on previously used questions was intended to re-examine earlier findings and facilitate cross-validation with the World Values Survey, the ISSP, the European Social Survey, and the Eurobarometer. At the same time, newly developed questions were designed to explore dimensions central to the project that have been absent in previous studies, such as global inequality and global redistribution, individual position in the global distribution, perceived social mobility, and perceptions of discrimination against immigrants.

### 3.2 Experimental design

In addition to general survey questions, the dataset contains data from six survey experiments. Three types of experiments were implemented: vignette experiments, conjoint experiments, and information treatment experiments. In vignette experiments, respondents evaluate fictional scenarios described in short vignettes that vary randomly across key characteristics (Auspurg and Hinz, 2015; Mutz, 2011). The aim is to identify which dimensions matter most to respondents and the relative impact of each. Conjoint experiments similarly estimate respondents’ preferences by asking them to evaluate alternative profiles that vary in their characteristics and are often presented in tabular form (Green and Rao, 1971). Information treatment survey experiments (Haaland et al., 2023) assess how exposure to specific information or framing influences perceptions of inequality and potentially shifts preferences for redistribution, social policies, and attitudes toward various social groups.

The experiments were conducted on survey subsamples organized into subject-specific modules. Four experiments were administered to separate subsamples of 2,000 respondents each, while two unrelated experiments were administered to the same subsample of 2,000 respondents. All experiments were pre-registered on the Open Science Framework (OSF).

The IneqPer survey included the following experiments:

*Poverty and redistribution preferences*: This information treatment experiment assessed how individuals’ attitudes toward redistribution are affected by exposure to information about their own relative poverty levels and their position in the income distribution relative to the poverty line. It explored whether personal experience and increased awareness of one’s own interest in redistribution shape support for redistributive policies.^3^

*Migrant children’s share and threat perceptions*: This information treatment experiment investigated whether presenting objective data on the proportion of children with migrant backgrounds in Italy affects public perceptions of migration related threats and attitudes toward immigrants.^4^

*Gender gap and fairness*: The gender pay gap experiment was a factorial survey (vignette) study exploring perceptions of fair wages and individual suitability for a job based on various characteristics. It investigated how factors such as age, ethnicity, parental status, field of study, occupation, and hobbies interact with an individual’s gender to influence respondents’ evaluations of ideal salary and job suitability.^5^

*Perceptions and normalization of gender-based violence*: This factorial survey (vignette) experiment assessed how different situations, which varied randomly across dimensions, are perceived in terms of the acceptance of gender-based violence and the likeliness of bystander intervention. It aimed to identify both societal and individual acceptance of ambiguous or more subtle forms of gender-based violence and the situational factors that affect the likelihood of intervention.^6^

*Perceptions of asylum seekers and refugees*: This image-based vignette experiment examined how respondents’ attitudes change based on the characteristics of asylum seekers depicted in photographs, which varied randomly across key dimensions. It investigated how these features influence assessments of perceived vulnerability, asylum claims, and support for government measures such as financial assistance, housing, and Italian language courses, which can facilitate the integration of asylum seekers.^7^

*Climate policy preferences and perceptions of climate fairness in Italy:* This conjoint experiment investigated public support for climate mitigation policies and perceptions of their fairness. It explored how respondents’ ideology (e.g., egalitarian orientations), trust in institutions, and perceived proximity to climate change impacts shape preferences for policy packages and their perceived fairness.^8^

### 3.3 Survey implementation and administration

The survey was conducted by the survey agency Dynata, and data were collected in two waves: from November 20 to December 19, 2024, and from February 15 to March 4, 2025. Quotas for gender, age, geographical area, education, and employment were strictly enforced across the entire sample as well as within each subject-specific experimental module. Respondents were randomly assigned to the specific modules while maintaining the original quotas, ensuring comparability among groups and subgroups in terms of sample distribution.

The response rate was 93%. Respondents were screened at the outset through questions regarding consent for data processing and a disclaimer on sensitive topics. Dynata employs a fully automated, multi-point quality control system called ‘QualityScore’ to assess respondents’ data and determine if they should be included in the final data set.^9^ A total of 1,292 cases were excluded due to low-quality scores, and approximately 2,840 respondents dropped out during the data collection process. The survey received ethical approval from the bioethics committee.

## 4 Core themes of the survey

### 4.1 Socio-economic inequality

Increasing socio-economic disparities have been evident over the past 30 years, including wage, income, and wealth inequality (Piketty, 2014). But to what extent are people aware of these sweeping changes? Are they well-informed about national and global levels of inequality? Equally important is understanding whether people perceive their society as equal or unequal (e.g., whether the majority of people are seen as living at the top or bottom of the society) and how they position themselves along the income distribution (Bellani et al., 2021). Perceptions of income inequality in the IneqPer survey include assessments of within-country inequality for both individuals and society, as well as assessments of global inequality. What follows are some selected findings from this section.

**Figure 1:**
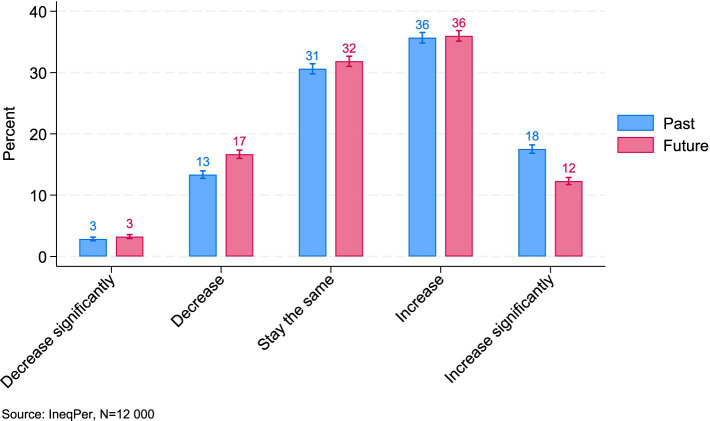
Perceived trends of income inequality (95% ci).

First, the perceived trend of income inequality in Italy was compared with the expected trend. Figure 1 illustrates Italians’ perceptions of these trends, showing their estimations of how inequality has evolved in the past and how they anticipate it will develop in the future. Respondents were asked whether income inequality has stayed the same over time, worsened, or improved and whether they expect changes in the future. The results suggest a prevalent perception that inequality in Italy has increased or significantly increased since 2000: about half of all respondents (54%) believed that inequality is getting worse. A similar share (48%) held pessimistic views about the future, anticipating that this trend of increasing inequality will continue. About a third of respondents believed that the situation in Italy has not changed since 2000, and approximately the same number thought that, in the future, inequality will remain at its current level. Overall, these findings suggest that there is little optimism for the future, with a large portion of the population expecting income inequality to persist or worsen in Italy (approximately 80%).

**Figure 2:**
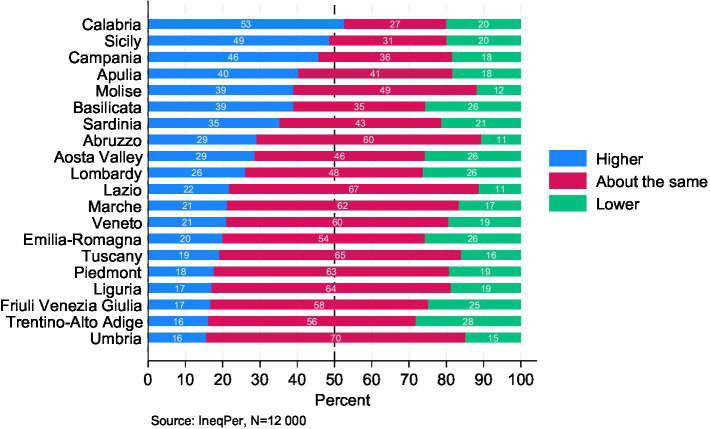
Perceived income inequality in different Italian regions compared to Italy as a whole.

In addition to perceptions of the overall situation and expectations regarding income inequality in the country, the IneqPer survey sheds light on how respondents evaluate the level of income inequality in their region compared to Italy as a whole (Figure 2). Responses varied across regions, reflecting significant differences in perceptions. In most southern regions, respondents perceived income inequality as higher than in Italy overall. Almost half of the respondents in Calabria, Sicily, Campania, Apulia, Molise, and Basilicata believed that inequality in their region exceeded the national average (53, 49, 46, 40, 39, and 39%, respectively). In contrast, residents of the northern and central regions generally perceived the level of inequality in their area to be comparable to the national average in Italy. In these regions, more than 50% of respondents shared this view, while the proportion of those who perceive income inequality as either significantly higher or lower was approximately half that figure.

**Figure 3:**
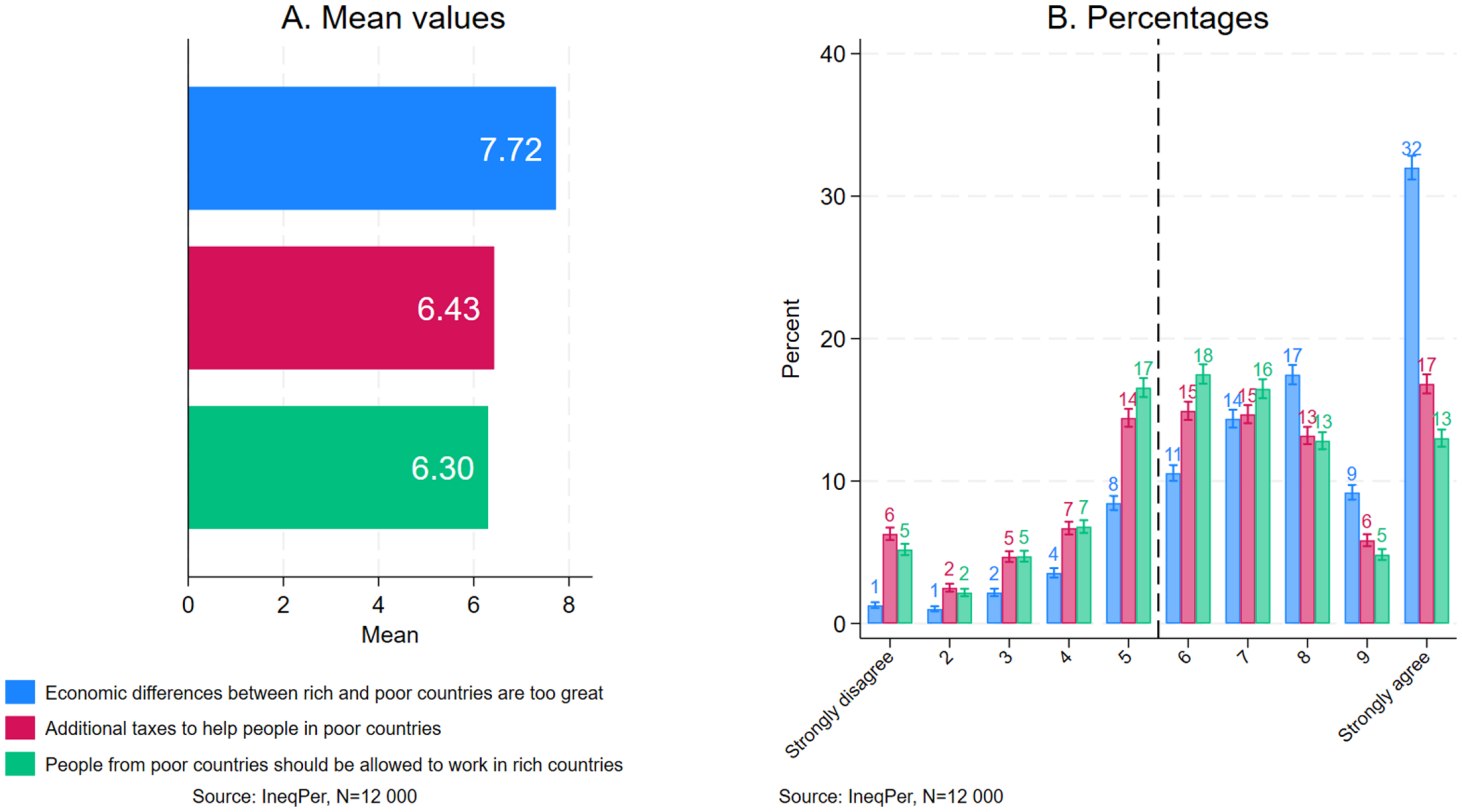
Attitudes toward global inequality: **(a)** mean values and **(b)** percentages.

Individual perceptions of global income inequality constitute another important focus of the IneqPer survey. The survey explored public attitudes toward global inequality and potential measures to address it. To this end, respondents were asked to indicate—on a 10-point scale ranging from 1 (strongly disagree) to 10 (strongly agree)—their level of agreement or disagreement with the following statements: ‘Differences between rich and poor countries are too large’, ‘People in rich countries should contribute additional taxes to help people in poor countries’, and ‘People from poor countries should be allowed to work in rich countries’. Figure 3 includes panels A and B: the first shows mean values for each statement, while the second shows the distribution of responses.

When asked about global economic disparities, respondents generally agreed that economic differences between rich and poor countries are too large, with a mean score of 7.72. While respondents acknowledged the significant economic gap between rich and poor countries, their support for policy measures aimed at mitigating this situation was a bit less pronounced. When asked about redistributive measures, such as additional tax contributions to support poorer countries, respondents showed moderate support, with a mean value of 6.43. Similarly, with respect to the liberalization of immigration and labor mobility as another potential policy response to global inequality, respondents also expressed moderate support: the mean score of 6.39 suggests a cautious endorsement of migration as a mechanism to address global inequality, reflecting a balance between openness and concern.^10^

Panel B in Figure 3 offers further detail on public perceptions of global inequality. About 32% of respondents strongly agreed (a score of 10) that economic differences between rich and poor countries are too large, while 17% strongly agreed that people in rich countries should pay additional taxes to support poorer countries, and 13% strongly agreed that people from poorer countries should be allowed to work in rich countries. Between 1 and 6% of respondents strongly disagreed with these statements, respectively. When respondents were grouped based on whether they generally agreed (scores from 6 to 10) or disagreed (scores from 1 to 5) with these statements, the patterns became even clearer. A substantial majority, approximately 84%, expressed support for the statement that ‘Differences between rich and poor countries are too large’. However, support for the second and third statements—‘People in rich countries should contribute additional taxes to help people in poor countries’ and ‘People from poor countries should be allowed to work in rich countries’—was lower, with roughly 65% of respondents reporting marginal or strong agreement with each statement. When considered together, the proportions and averages provide a complementary understanding of the responses. For instance, substantial support of the statement that economic differences between rich and poor countries are too large, as indicated by high mean scores, mostly stems from the ‘strongly agree’ responses.

In general, the findings presented in Figure 3 highlight a significant divide in public opinion: while a substantial share of people supported measures aimed at reducing global inequality, like additional taxes in favor of poor countries or more liberal labor migration policies, nearly a third of respondents were opposed to such measures. Taken together, these findings point to a complicated public perception of global inequality: although there is widespread recognition of the problem, support for specific policy measures to address it is more fragmented, reflecting the complex and often polarizing nature of these issues.

**Figure 4:**
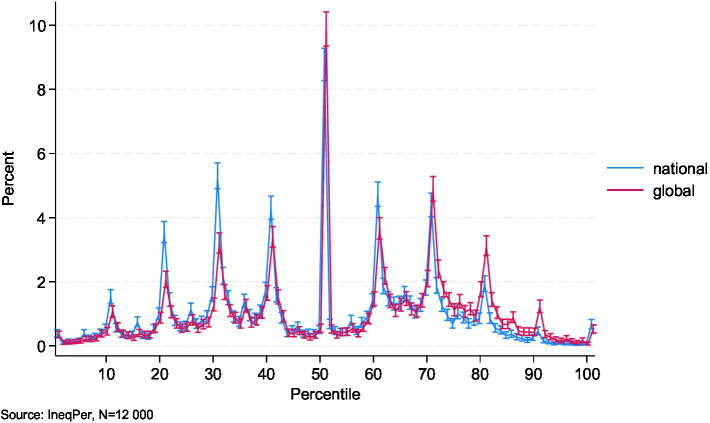
Perceived income ranks in national and global income distributions.

The IneqPer data also provide insights into how respondents perceive their position in both the national and global income distribution (Figure 4). These results highlight a tendency for people to view themselves as higher up in the global income distribution than in the national one, although the gap between global and national perceptions varied along the distribution. Around 8–10% of respondents saw themselves as positioned precisely in the middle of the income distribution or immediately around it, while fewer than 2% of Italians in each group saw themselves at the very top or bottom, whether at the national or global level. Notably, at the 30th and 40th percentiles, as well as around the 60th percentile, individuals tended to perceive themselves as higher in the national income distribution than in the global one.

When we divided respondents based on their perceived rank relative to the 50th percentile in the national income distribution, 47% placed themselves below the midpoint, 9% located themselves exactly at the midpoint, and 44% placed themselves above it. At the same time, perceptions of individual positions in the global income distribution were more optimistic: 37% of respondents perceived themselves below the global median, 10% at the median, and 53% above it. These findings suggest a clear upward shift in perceptions when individuals consider their position on a global scale rather than within Italy.

The detailed summary statistics for perceived positions in national and global income distributions reveal some interesting findings. For the national income distribution, the median self-placement is at the 50th percentile, with a mean of 48.36 (SD = 21.65), indicating that, on average, respondents perceived themselves to be near the middle of the national income spectrum. The interquartile range suggests that half of the respondents placed themselves within this broad central part. At the extremes, fewer than 1% of respondents placed themselves below the 5th percentile or above the 95th percentile. At the same time, Figure 4 shows that a large share of respondents located themselves around the 30th and between the 60th and 70th percentiles in the national income distribution.

By contrast, in the global income distribution, respondents perceived their positions more favorably. The median self-placement rises to the 57th percentile, with a mean of 53.53 (SD = 22.31), suggesting that respondents, on average, viewed themselves as better off in the global income hierarchy than within the national one. The interquartile range for global self-placement (36th to 71st percentiles) is shifted upward compared to the national distribution.

### 4.2 Gender inequality

Gender inequality in society has been widely documented despite past progress in highly developed countries. While women have made notable strides toward equality in education and human rights, gender inequalities persist in labor market outcomes and access to prestigious occupational positions (England, 2010), as well as in political representation (Kenworthy and Malami, 1999). However, how do individuals perceive both the progress that has been made and the ongoing challenges faced by women in society? Several survey items focused on addressing this question, including perceptions of current gender inequality overall in Italy and in a comparative perspective by comparing the overall gender inequality with those of regions, and perceptions of change over time.

**Figure 5:**
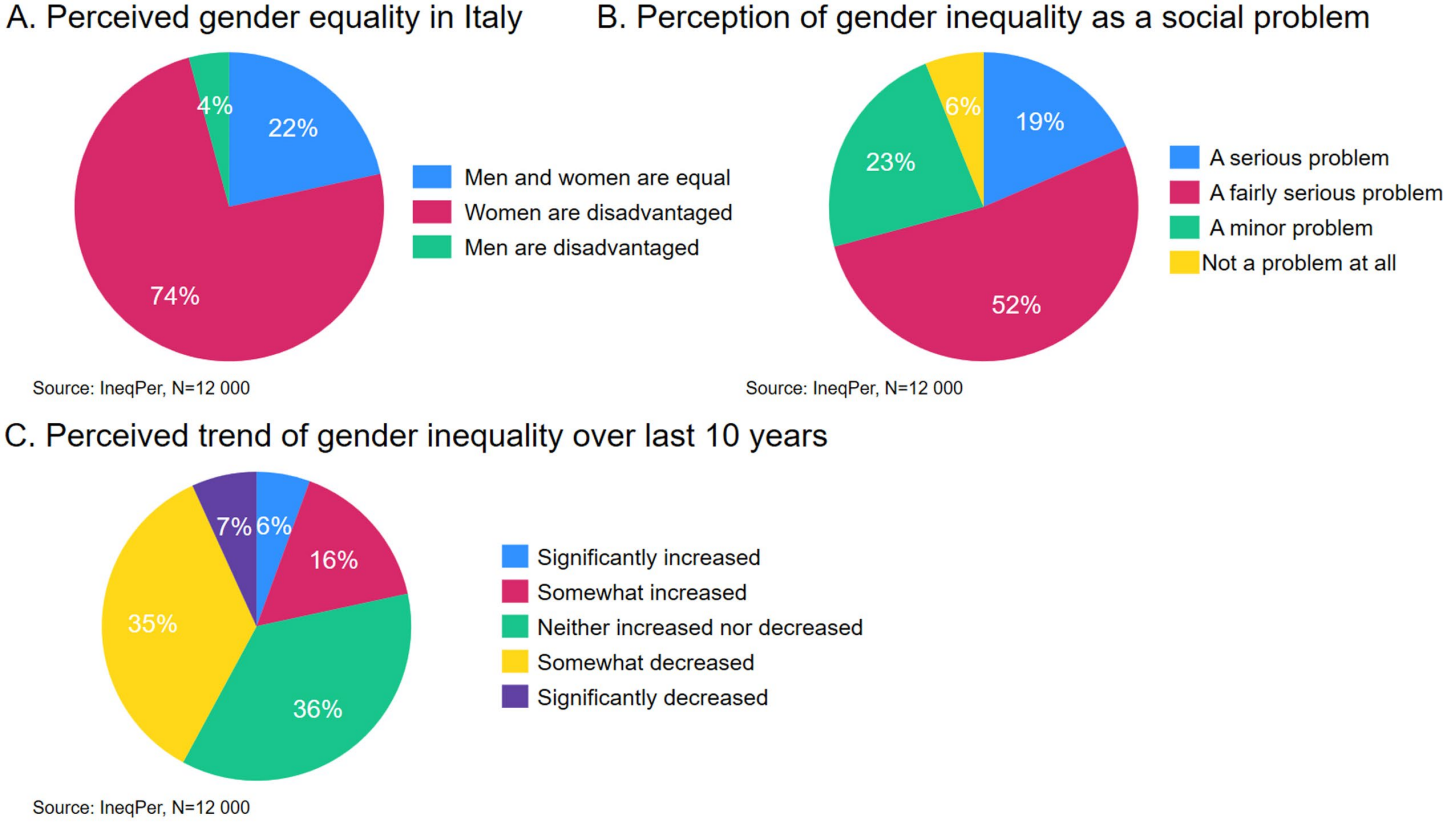
Perceptions of gender inequality: **(A)** Perceived gender equality in Italy, **(B)** Perception of gender inequality as a social problem, **(C)** Perceived trend of gender inequality over the last 10 years.

First, the survey asked respondents whether men and women are treated equally in Italy (Figure 5). A small portion of respondents (22%) agreed with this statement. By contrast, most respondents (74%) believed that women are disadvantaged compared to men, while a small share (4%) felt that men are disadvantaged compared to women. Respondents were also asked to evaluate whether inequalities between men and women constitute a serious social problem in Italy. For the majority, gender inequality was considered a significant issue, with 71% considering it to be a ‘serious’ or ‘fairly serious’ problem. A smaller proportion (23%) viewed gender inequality as a minor problem, and a minority (6%) did not perceive it as a problem at all. Overall, these findings suggest that gender inequality is broadly recognized as a serious or fairly serious issue in Italy.

Perceptions of change over time provide a more nuanced picture. When asked about the change in gender inequality over the past decade, over one-third of respondents (36%) indicated that inequalities between men and women neither increased nor decreased. A similar proportion (35%) felt that inequalities somewhat decreased, while 7% thought they had significantly decreased. The remaining 22% of respondents felt that inequalities had increased. Thus, even though gender inequality is considered an important problem, there is an overall impression that inequalities either decreased or stayed the same over time.

**Figure 6:**
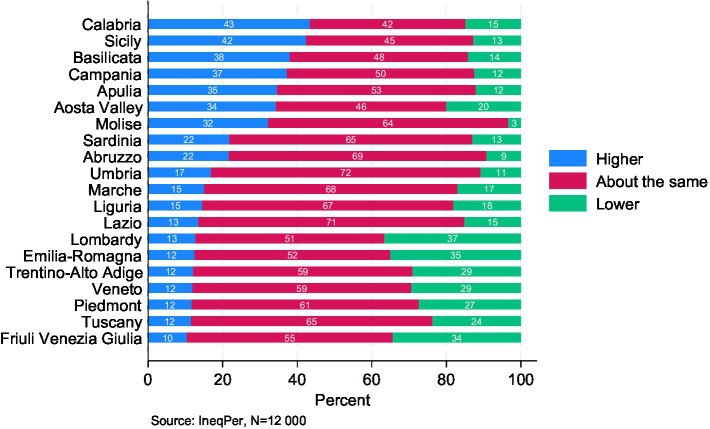
Perceived gender inequality in different Italian regions compared to Italy as a whole.

Respondents were also asked to assess the level of gender inequality in their own region compared to the national average (Figure 6). Most respondents (57%) felt that the level of gender inequality in their region was about the same as the national average. However, there were notable regional differences. Respondents from northern regions were more likely to perceive lower levels of gender inequality in their regions compared to the national average, while respondents in the south were more likely to perceive higher gender inequality in their regions compared to Italy as a whole. For example, Calabria (43%), Basilicata (38%), and Sicily (32%) had the highest proportions of respondents who perceived gender inequality in their region as higher than the national average. In contrast, only 10% of respondents in Friuli-Venezia Giulia and 12% in Emilia-Romagna, Veneto, Trentino-Alto Adige, Piedmont, and Tuscany saw gender inequality in their regions as higher than the national average.

### 4.3 Perceptions of migrants in Italy

A prominent feature of the foreign population in Italy is its considerable heterogeneity with respect to countries of origin. According to the latest official statistics (Istat, 2024),^11^ more than 5 million non-Italian residents originate from 194 distinct nations, posing substantial challenges to effective integration. The most common countries of origin are predominantly located in Eastern Europe, accounting for 42% of the total migrant population, followed by North Africa and Southeast Asia, each representing 13%. In contrast, countries from other regions contribute only marginally to the overall composition.

**Figure 7:**
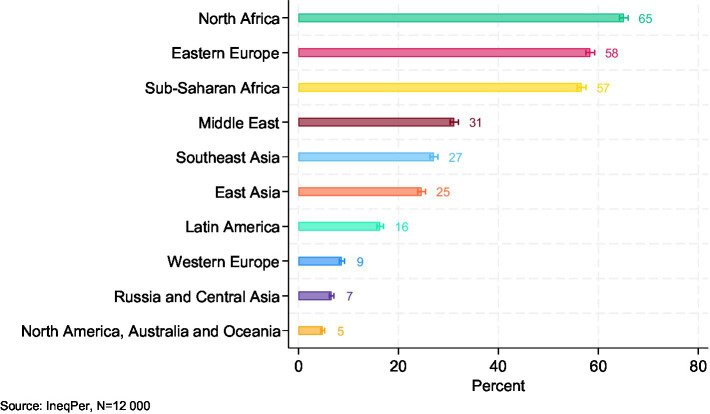
Perceived countries of origin of immigrants; multiple choice of up to three regions (95% CI).

To understand how Italians perceive the origins of immigrants, respondents in the IneqPer survey were asked to identify up to three perceived regions of origin for immigrants in Italy. The responses reveal an awareness of the diversity of Italy’s foreign population, albeit with some misconceptions (Figure 7). North Africa was the most frequently mentioned region, identified by 65% of respondents as a source of immigration. This was followed by Eastern Europe (58%) and Sub-Saharan Africa (57%). Smaller shares of respondents mentioned the Middle East (31%), Southeast Asia (27%), and East Asia (25%) as regions of origin. Fewer associated immigration with Latin America (16%), Western Europe (9%), and Russia and Central Asia (7%). The lowest perceived share was for North America, Australia, and Oceania, as mentioned by just 5% of respondents.

It is widely documented that migrants in most national contexts do not have equal access to opportunities, often lagging behind natives in overall achievement, education, and particularly in the labor market (Diehl et al., 2016; Huddleston et al., 2015). The reasons for these inequalities vary and may result from structural factors, such as limited proficiency in the host language, overrepresentation in low-skilled and precarious work, generally lower levels of formal education, difficulties in recognizing foreign qualifications, and barriers to social integration, which is often exacerbated by prejudice and discrimination (Diehl et al., 2021). But are these reasons adequately considered by citizens? How do citizens see migrants and their position in society?

Against this backdrop, this section examines items and their descriptive statistics regarding perceptions of immigrants and the discrimination they face in Italy. The survey included questions on stereotypes about migrants using a 7-point semantic differential slider, drawing on established work on semantic space and stereotype content (Osgood, 1964; Morland and Williams, 1969; Fiske et al., 2002). The semantic-differential instrument integrates four key stereotype dimensions. First, it draws directly on the Stereotype Content Model for core *warmth* items: not sincere–sincere, hostile–friendly, cold–warm (Fiske et al., 2002). Second, it follows the traditional semantic differential method to measure strength, using the pair weak–strong (Osgood, 1964; Back et al., 1972). Third, combining these approaches, it assesses *competence* with items such as unconfident–confident, incompetent–competent, unambitious–competitive, and subservient–independent (Fiske et al., 2002; Benard et al., 2023). Fourth, guided by mixed-method adaptations, it measures an *activity/competence* dimension with dangerous–harmless bipolar adjectives (Jenaro et al., 2018). Finally, to address everyday interactional and work-ethic stereotypes, two purely semantic differential-based extensions—rude–polite and lazy–hardworking—were added.

In addition to evaluating stereotypes about migrants, respondents were asked to apply the same set of questions to Italians. This allowed a direct comparison between their perceptions of migrants and natives, ensuring a comprehensive assessment of perceptions across the main stereotype dimensions. Respondents were asked to evaluate the majority of migrants using the bipolar adjectives described above, with the instruction: ‘Think about the majority of foreigners residing in Italy and use the slider to evaluate them based on the following traits’. The 11 bipolar adjective pairs were presented in random order, with participants responding on a seven-point scale evenly spaced between each pair of antonyms. The mid-point of the scale indicated neutrality (0).

**Figure 8:**
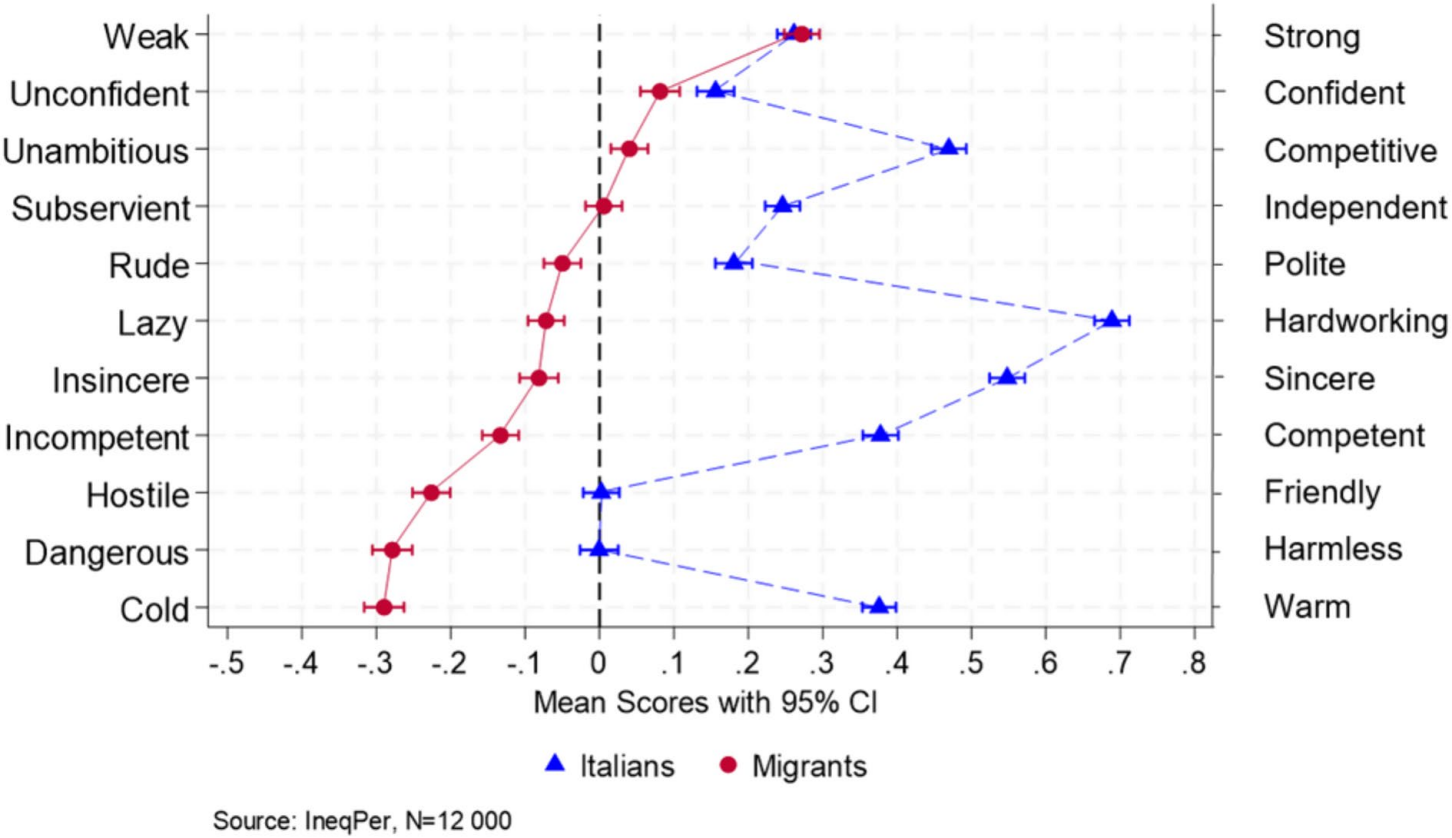
Perceptions of immigrants and natives in Italy (95% CI).

Figure 8 compares perceptions of migrants and natives across 11 bipolar adjectives. Across all three items measuring warmth, natives were generally perceived more favorably, being rated as friendlier and warmer compared to migrants. While respondents rated natives as neutral on the insincere–sincere scale, this evaluation was more positive than the corresponding rating for migrants. Competence-related traits also revealed notable disparities in the perceptions of migrants and natives. Native Italians were perceived as more competent, confident, competitive, and independent. The sole exception was the unconfident–confident item, where migrants received a neutral score; for all other competence-related items, migrant scores fell below zero.

The activity dimension, assessed through the dangerous–harmless item, also indicates unfavorable perceptions of migrants. As in previous cases, migrants received an average score below zero, compared to scores above zero for natives. Regarding other features, while migrants were rated lower on politeness, they received scores comparable to natives for the lazy–hardworking item. The only other dimension where immigrants were rated similarly to natives was potency, measured by the weak–strong item. Both migrants and natives received scores above zero, with no statistically significant difference, indicating that both migrants and natives were perceived as relatively strong.

The averages for all 11 items ranged between −0.5 and 0.5, suggesting a relatively small gap in perceptions of Italians and immigrants. Nevertheless, the discrepancies remain statistically significant, with most average scores for migrants falling below zero and those for natives above zero. These patterns across nearly all dimensions point to unfavorable stereotypes against migrants, potentially reflecting underlying biases related to social integration.

**Figure 9:**
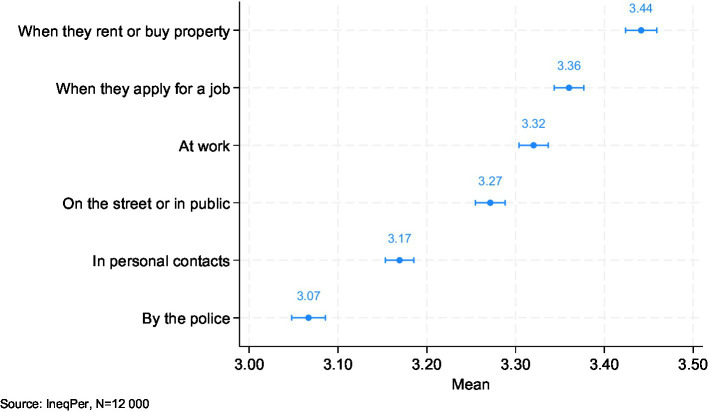
Perceived discrimination of immigrants (95% CI).

Respondents were also asked to evaluate the perceived discrimination faced by immigrants on a 5-point scale measuring how often the majority of immigrants experience discrimination because of their ethnicity (ranging from 1 ‘never’ to 5 ‘always’). The assessment covered six dimensions reflecting various aspects of social and economic life, including the labor market, housing, and personal interactions. The data indicate a generally moderate perception of discrimination across multiple areas (Figure 9). The highest reported level of perceived discrimination was in the context of renting or buying property, with a mean score of 3.44. This was closely followed by discrimination perceived in job applications (3.36). Discrimination experienced in public spaces, such as on the street, was also evaluated as moderate (3.32). In terms of personal interactions, respondents reported slightly lower levels of perceived discrimination, while discrimination by the police was seen as the least common (3.07). These findings stress that perceived discrimination is most prominent in housing and employment situations, with public spaces and personal interactions also being areas of concern. However, discriminatory practices by law enforcement were considered less frequent.

## 5 Cross-validation

Cross-validation checks were conducted on selected variables, including political orientation, experiences of discrimination, gender attitudes, and socio-economic inequality. Cross-validation of the IneqPer results with established benchmarks from reputable international datasets for Italian samples ensures the reliability and validity of the survey findings. For this purpose, data from three recent international surveys were used: the European Social Survey (ESS10, 2020), the European Value Study [Joint EVS/WVS 2017–2022 dataset (EVS/WVS, 2017)], and the International Social Survey Program (ISSP Research Group, 2019).

### 5.1 Political attitudes, discrimination, and gender attitudes

First, respondents’ political attitudes in the IneqPer survey were compared with those of the ESS10 (2020) survey. Both surveys measure political orientation using a left–right scale ranging from 1 (‘left’) to 10 (‘right’). The results from both surveys indicate a similar pattern, showing that respondents in both samples were generally positioned near the center of the political spectrum. However, there is a slight difference in the mean values: the IneqPer survey reports a mean of 5.53, while the ESS10 survey shows a slightly lower mean of 5.13. This small difference may indicate that the IneqPer sample was marginally more right-leaning compared to the ESS10 sample. Alternatively, it might reflect a modest rightward shift of the Italian population during the five-year gap between the surveys.

**Figure 10:**
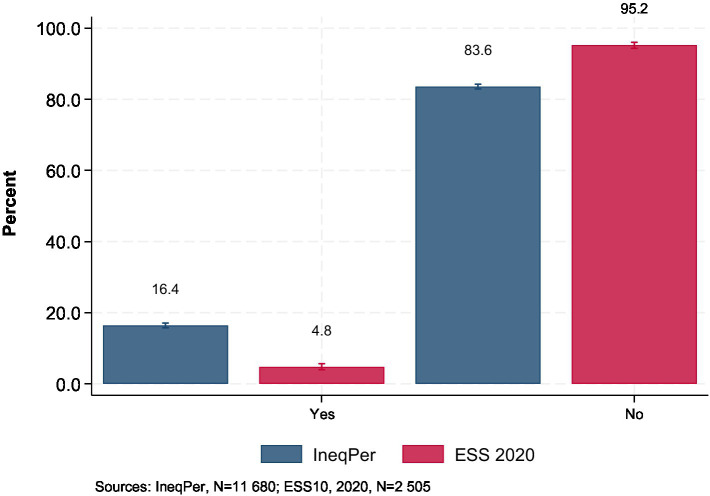
Perception of being a member of a group that is discriminated against (95% CI).

The IneqPer survey also addresses perceptions of personal discrimination and the reasons for this perceived discrimination. The questions ‘Would you describe yourself as being a member of a group that is discriminated against in this country?’ and ‘On what grounds is your group discriminated against?’ were derived from ESS10. As shown by Figure 10, in the IneqPer survey, 16% of respondents reported themselves as members of a group that faces discrimination, while the majority, 84%, did not feel they belonged to such a group^12^. This finding highlights that while discrimination was perceived to be rare by the sample, it remains a significant concern for a minority. In the ESS10, 5% of respondents reported experiencing discrimination, compared to 95% who did not. This difference, as in the previous case, may also reflect changing public perceptions over time.

**Figure 11:**
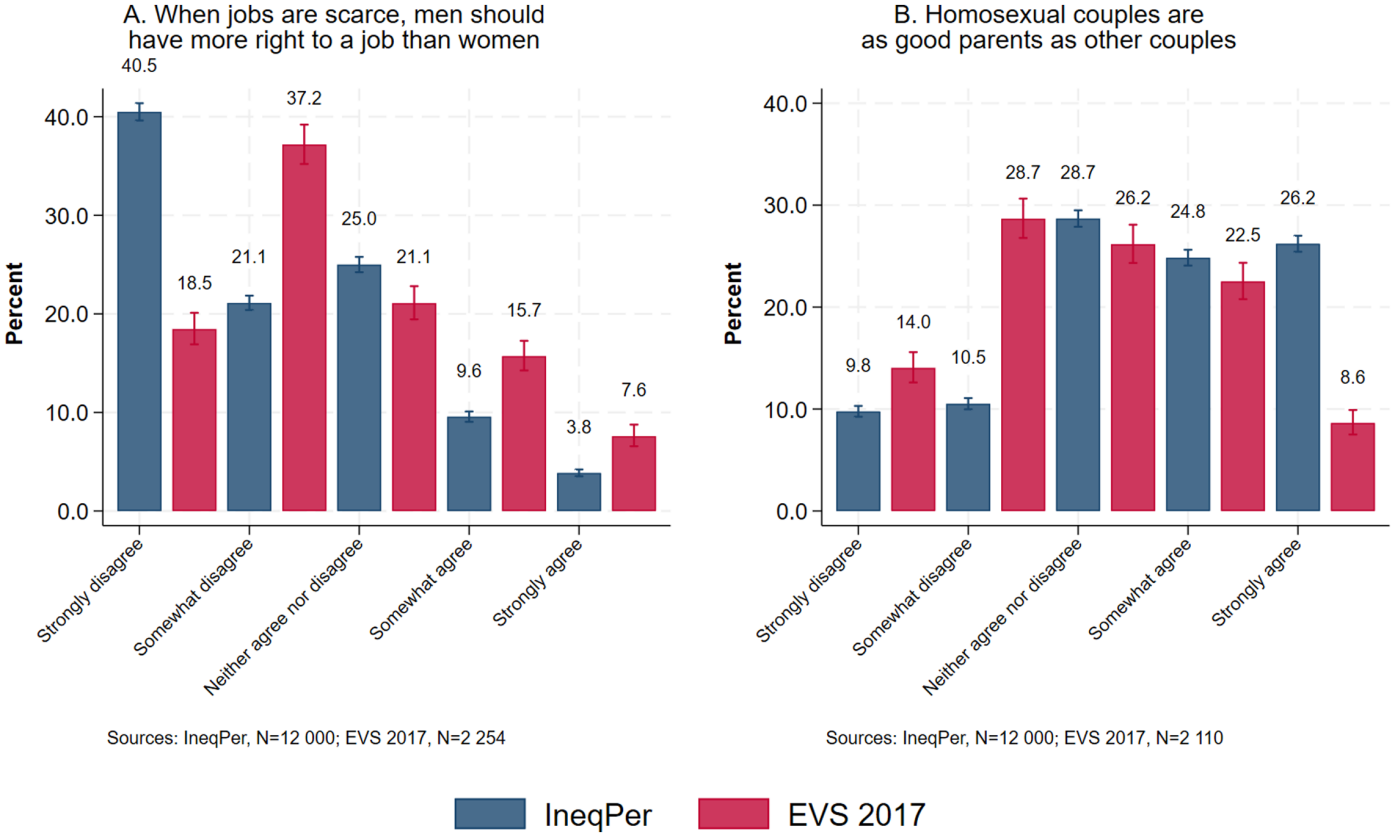
Gender attitudes: **(A)** When jobs are scarce, men should have more right to a job than women, **(B)** Homosexual couples are as good parents as other couples (95% confidence intervals).

The IneqPer survey also examined various dimensions of gender attitudes using a 5-point scale, where 1 means ‘strongly disagree’ and 5 ‘strongly agree’. The questions were derived from the European Value Study (2017) or adapted from other surveys. Specifically, the statements ‘When jobs are scarce, men have more right to a job than women’ and ‘Homosexual couples are as good parents as other couples’ were adopted in identical form from the EVS. The cross-validation results (Figure 11) for the first item show that 40.5% of IneqPer respondents strongly disagreed, and 21.1% somewhat disagreed with the statement, compared to 18.5 and 37.2% in the EVS. Additionally, less than 5% of IneqPer respondents strongly agreed with the statement, compared to 7.6% in the EVS. Regarding the second question on homosexual couples (Figure 11), 26.2% of IneqPer respondents agreed, and the same percentage strongly agreed with the statement, whereas in the EVS, only 8% strongly agreed and 22.5% agreed. Both comparisons suggest that the IneqPer dataset contains a larger percentage of respondents with less traditional attitudes compared to the EVS.

### 5.2 Current perceived and desired shape of inequality and important factors for getting ahead in life

**Figure 12:**
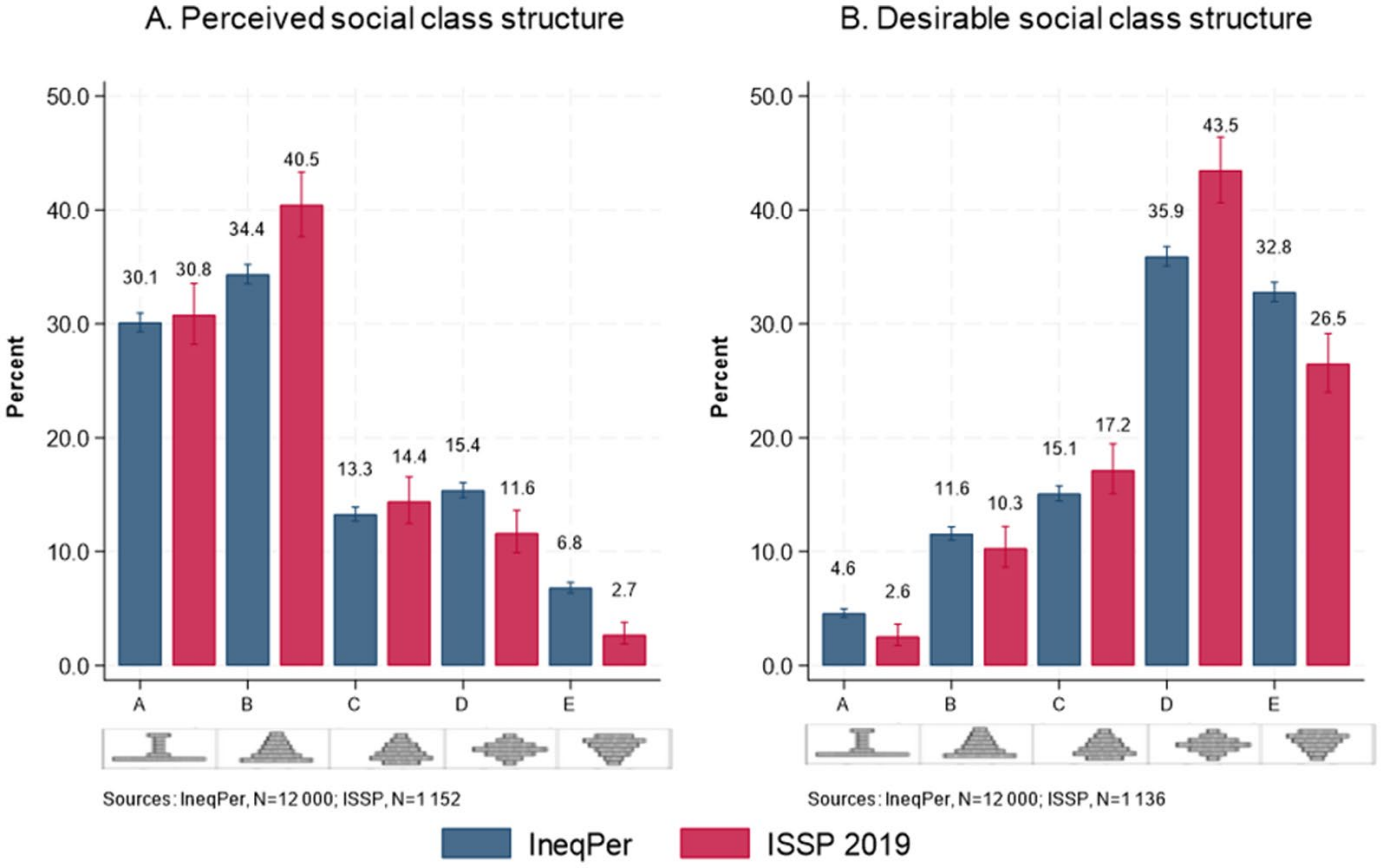
Perceptions of social class structure: **(A)** Perceived social class structure, **(B)** Desirable social class structure.

A comparison between perceived and desirable social class structure between the IneqPer 2024 and ISSP 2019 surveys shows substantial similarities (Figure 12). When asked to identify the current shape of society in terms of inequality in Italy, IneqPer respondents were presented with five diagram types representing various societal structures. Type A shows a small elite at the top, very few people in the middle, and the vast majority at the bottom; type B shows a society shaped like a pyramid, with a small elite at the top, more people in the middle, and the majority at the bottom; type C is a pyramid, except that only a few people are at the bottom; type D is a society where the majority of people are in the middle; and type E depicts a society with many people near the top and only few at the bottom. The most common response, representing 34% of the sample, was Type B. This suggests that a significant portion of the population perceives Italy as having a relatively large middle class but with an unequal distribution where a small elite dominates. Following this, type A was selected by 30% of respondents, indicating a more pronounced inequality with a small wealthy class and a majority at the lower economic levels. Type D, depicting a society where most people are in the middle, was chosen by 15% of the respondents, while Type C was selected by 13%. Finally, only 6.83% of respondents identified Italy as resembling Type E, a society with many people near the top and only a few near the bottom.

When asked how Italy should ideally look in terms of inequality, the preferences of respondents shifted significantly. The most popular choice, selected by 36%, was Type D, indicating that most respondents desire a more equitable society where most people are in the middle, reducing disparities between the wealthy and the poor. This analysis suggests that while the current perceived shape of inequality in Italy leans toward more pronounced inequalities (as reflected in the higher number of respondents selecting Types A and B), there is a strong preference for a more equitable society, with respondents favoring Types D and E as the ideal structure.

In the ISSP, 31 and 40% perceived Italy as a Type A and Type B society, respectively, and 12 and 3% as Type D and Type E. Similarly, approximately 43% of respondents desired Italy to be a Type D and 26% desired Italy to be Type E.

**Figure 13:**
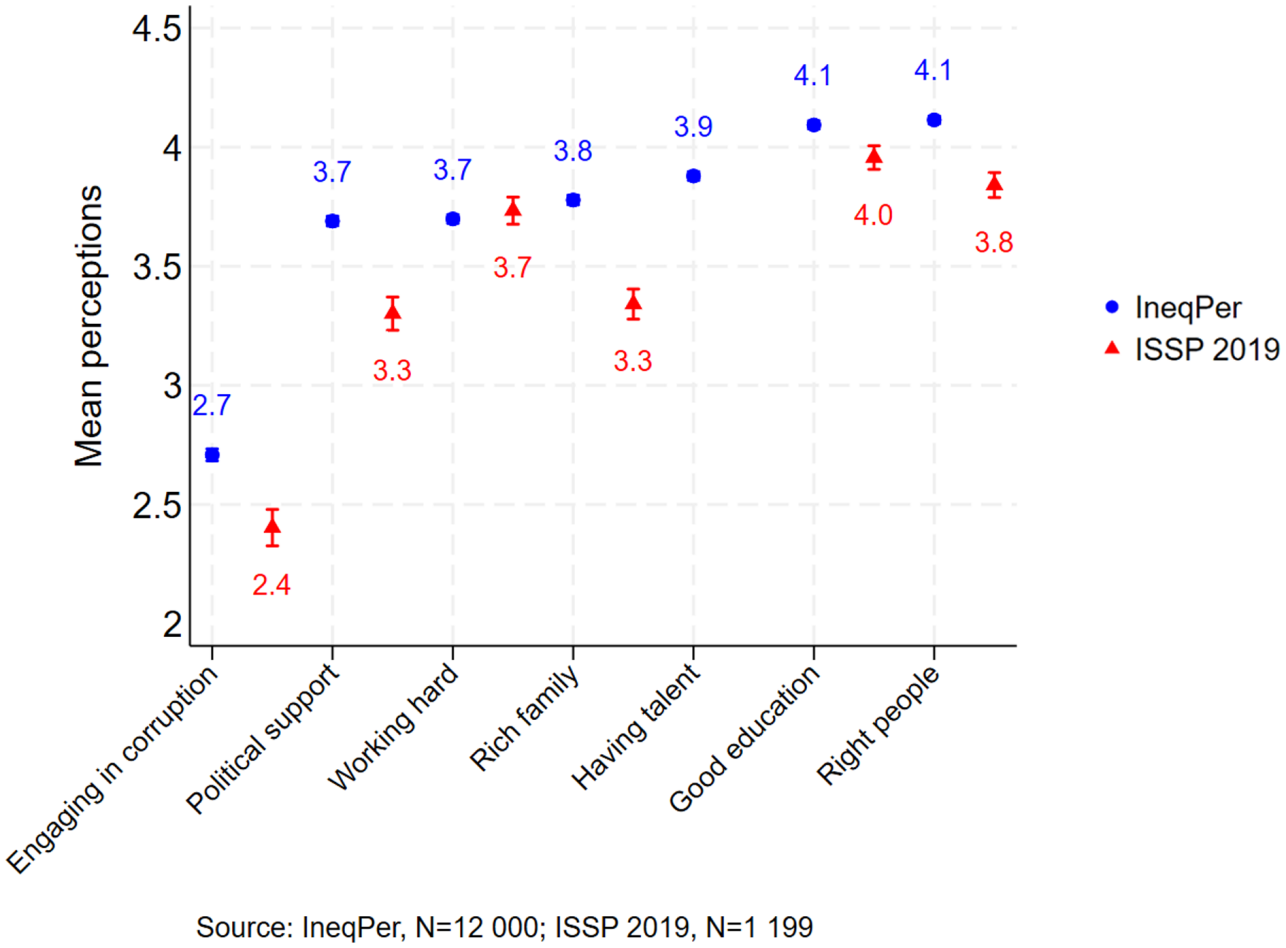
Factors for getting ahead in life (95% ci).

Both the IneqPer survey and the ISSP 2019 also examined respondents’ perceptions of the factors necessary to get ahead in life in Italy (Figure 13). Respondents evaluated various determinants, including ‘coming from a wealthy family’, ‘having a good education’, ‘working hard’, ‘knowing the right people’, ‘having political support’, and ‘paying bribes or engaging in corruption’. Responses were collected using a 5-point scale (Ranging from 1 = Essential to 5 = Not important at all), although in this paper, the scale has been reversed for the sake of comparability.

The IneqPer and ISSP 2019 data show similar patterns in perceived conditions of success, especially regarding factors like education and hard work. In both datasets, ‘having a good education’ and ‘working hard’ were rated as relatively important, with mean values of 4.09 and 3.7 for the IneqPer and 3.96 and 3.73 for the ISSP 2019, respectively. Additionally, both surveys noted the significance of ‘coming from a wealthy family’, with minor differences in ratings but consistently indicating this as a moderately important factor for success. Both surveys ranked ‘paying bribes’ as the least important determinant of success, with average scores of 2.40 in the ISSP and 2.71 in the IneqPer.

There were some differences between the two surveys, particularly regarding factors such as wealth, work ethic, political support, and corruption. Respondents to the IneqPer survey placed greater emphasis on ‘knowing the right people’ (mean: 4.11) and ‘coming from a rich family’ (mean: 3.78) compared to the ISSP 2019 (means: 3.84 and 3.34, respectively). Overall, while differences in mean values between the two datasets exist, they were relatively limited and suggest consistent findings, thus supporting the external validity of the IneqPer survey.

## 6 Implications and conclusion

The article is structured around a large-scale cross-sectional survey and several experimental interventions aimed at understanding the determinants of inequality perceptions, their consequences for public opinion, and potential methods to challenge misperceptions. It has described the content of the survey and the process of data collection. It has also provided a summary of some of the major variables. Finally, cross-validation of the survey was assessed by comparing its findings with a range of recent international datasets. The dataset (*N* = 12,000) may contribute to the development of policies aimed at addressing inequality, fostering social inclusion, and improving public understanding of inequality.

The initial results show that Italians indeed recognize inequality across multiple dimensions (e.g., socio-economic inequality, gender inequality, and inequality between migrants and natives), yet they do not seem to strongly perceive that they are a subject of discrimination. Italians seem to desire a more equitable society in terms of income differences, but they are not overtly optimistic about positive changes in the future. They also recognize the extent of global inequality, though they are only modestly in favor of some form of global redistribution of advantage. Their perceptions of their own position in both the global and national income distribution largely overlap. Similarly, Italians acknowledge the existence of gender inequality, and unlike their rather pessimistic outlook on income inequality, they perceive some positive progress over time in this area. Regional differences were also noted, with significantly more respondents from the south of Italy, compared to those in the north, evaluating both socio-economic and gender inequalities as higher in their regions compared to the national average. Conversely, a larger number of respondents from the north evaluated inequality as comparatively lower than the national average. The results also show bias regarding migrant population. Immigrants were perceived more negatively in terms of social traits, suggesting the influence of societal stereotypes on individual attitudes. Discrimination against migrants was perceived as frequent, particularly in housing and the labor market.

Do these perceptions reflect the reality of inequalities in Italy? It may be argued that the perceptions collected by the IneqPer survey indeed align with some of the objective indicators. For instance, socio-economic inequality was recognized as a salient issue without optimism for improvement—similar to the objective indicators noted in the introduction (World Bank, 2025). Gender inequality was also seen as a serious concern, though citizens perceived at least some improvement over time, mirroring observed trends (EIGE, 2024). The IneqPer data also signal the presence of stereotypes and perceived discrimination against immigrants, consistent with current data (Amo-Agyei, 2020; Caneva, 2014).

While the survey design and experimental interventions aimed to be comprehensive, the use of a non-probability sampling method (quota sampling) introduced potential limitations in terms of generalizability. Moreover, although the cross-validation checks show substantial correspondence between the IneqPer data and other value surveys, respondents from the IneqPer survey tended to have slightly more progressive views compared to these other surveys. This difference could potentially be due to a time gap between the IneqPer and the other surveys, as the latter were conducted between 2018 and 2020, but also due to sampling or methodological differences. Unfortunately, more recent data are not available, and many of the IneqPer questions do not correspond to the available international surveys. Furthermore, the IneqPer survey may underrepresent certain segments of the population, particularly groups with lower educational attainment and the unemployed, though post-stratification weighting serves to mitigate some of these issues. Future research could consider potential extensions of the current survey to other geographical contexts and regions.

## Data Availability

The datasets presented in this article are not readily available because the dataset is used exclusively by internal project members. Requests to access the datasets should be directed to https://ineqper.unipv.it/.

## Footnotes

^1^ https://data.worldbank.org/indicator/SI.POV.GINI/

^2^Further analyses in the article relied on sample statistics and were not adjusted by weights.

^3^For further information, see: https://osf.io/84njk

^4^For further information, see: https://osf.io/bfz84

^5^For further information, see: https://osf.io/6q4f8

^6^For further information, see: https://osf.io/zbgh6

^7^For further information, see: https://osf.io/kuf4e

^8^For further information, see: https://osf.io/b4fgx

^9^All page data was collected and passed through a 20 + point ML model using a combination of passive data and survey responses to capture and isolate poor-quality respondents. For instance, QualityScore monitors respondents’ overall survey speed and flags those who complete questions too quickly or exhibit speed behaviors. QualityScore is determined through several collected in-survey data points that the tool utilizes in its ML scoring algorithm. If the question is a grid question with 5 + options, QualityScore flags each time a respondent answers the same exact score/rating for each option. It also monitors in-survey passive behavior like respondents’ mouse movement, activity/inactivity levels, and page resubmissions, among others, and flags anomalistic behaviors.

^10^Although the questions were derived from the ISSP, IneqPer utilizes different scales of measurement. For instance, the IneqPer survey employed a 10-point even scale that facilitated collecting more detailed information about the distribution of perceptions and avoiding the tendency of respondents to opt for a more central position. In contrast, the ISSP used a 5-point scale. For these reasons, a direct cross-validation was not applied.

^11^ https://demo.istat.it/?l=it

^12^320 respondents in the IneqPer survey (0.03%) preferred not to answer, and were excluded for the comparison between the datasets.
