# Correction: A comprehensive overview of a large-scale survey on inequality perceptions (IneqPer) in Italy

**DOI:** 10.3389/fsoc.2025.1688195

**Published:** 2025-09-02

**Authors:** Nevena Kulic, Olga Griaznova, Eleonora Clerici, Daniela Bellani, Debora Mantovani, Loris Vergolini, Francesco Scervini

**Affiliations:** ^1^Department of Political and Social Sciences, University of Pavia, Pavia, Italy; ^2^Department of Statistical Sciences, Catholic University of Sacro Cuore, Milan, Italy; ^3^Department of Political and Social Science, University of Bologna, Bologna, Italy

**Keywords:** inequality, perceptions, socio-economic status, gender, immigration, Italy, survey experiments

In the published article, information was omitted from the Funding statement.

The corrected statement reads: The author(s) declare that financial support was received for the research and/or publication of this article. This research was funded by Next Generation EU – under the Piano Nazionale di Ripresa e Resilienza PNRR – Mission 4 – Component 2 – Investment 1.1 “Fondo per il Programma Nazionale di Ricerca e Progetti di Rilevante Interesse Nazionale (PRIN)” (Ministerial Decree of the Ministry of University and Research (MUR) No. 1409 of 14/09/2022); PRIN 2022 PNRR research project entitled “Inequality between reality and perception: socio-economic status, gender and immigration in Italy (IneqPer),” Project Code P2022TWZN3_002, CUP J53D23017070001.

The original version of this article has been updated.

