# Supplementary material for: A comprehensive overview of a large-scale survey on inequality perceptions (IneqPer) in Italy
Source: Front Sociol. 2025 Jul 18;10:1620096. doi: 10.3389/fsoc.2025.1620096 (PMC12313601; doi:10.3389/fsoc.2025.1620096)
Supplement: Supplementary file 1 [file Data_Sheet_1.PDF]

IneqPer project QUESTIONNAIRE

INTRODUCTION 2
Socio-demographic characteristics and attitudes 3
Gender inequality 14
Income inequality 16
Migration and ethnic inequality 19
Global inequality 21
MODULE – Income and social status 22
MODULE – Gender gap and fairness 23
MODULE – Gender-based violence 24
MODULE – Global challenges 26
MODULE – Migration 28

INTRODUCTION

Dear Participant,

We invite you to take part in a research project conducted by the University of Pavia, in collaboration with the University of Bologna and the Catholic University of Milan, focusing on inequalities in Italy. Your participation is important to us, and your opinions are invaluable for gaining deeper insights into this issue. Your involvement in the research project will take approximately 20 minutes.

As it is your right to be informed about the purpose and characteristics of this research project, and to allow you to make an informed and voluntary decision to participate, we kindly ask you to carefully read the information sheet and the data privacy notice, which can be downloaded here:

DOWNLOAD INFORMATION SHEET<br>View the PDF “NUOVO Modulo Informativo sul trattamento dei dati”	☐

Please note that this research project has been approved by the Ethics Committee of the University of Bologna.

We thank you for your willingness to participate.

NEXT PAGE

In this survey, we would like to ask you some questions that may be considered sensitive, such as topics related to Religion, Politics, Sexual Orientation, Gender Identity and Violence, and Race/Ethnicity. Your responses to these questions are entirely optional, and you may withdraw your consent at any time. The responses you provide will be used exclusively for analysis and market research purposes.

Do you consent to the collection of this information?

For more details on how your information will be processed and protected, please review the Privacy Policy of the University of Pavia, in collaboration with the University of Bologna and the Catholic University of Milan, here:

POP-UP: View the PDF “NUOVO Modulo Informativo sul trattamento dei dati”

☐	Yes, I consent [CONTINUE]	To participate in the research project
☐	No, I do not consent [SCREEN OUT]	To the processing of my personal data necessary for participation in the research project.

Socio-demographic characteristics and attitudes

Q1. How old are you? ____________[1]

Q2. With which gender do you identify?

a) Female
b) Male
c) Other

Q3. Is this the sex you were assigned at birth?

a) Yes
b) No
c) Prefer not to answer

Q4. Are you a citizen of Italy?

a) Yes
b) No

IF Q4 = a (Citizen of Italy) → GO TO Q5
IF Q4 = b (NOT citizen of Italy) → GO TO Q6

Q5. Are you a citizen of Italy from birth?

a) Yes
b) No

IF Q5 = a (Citizen of Italy from birth) → GO TO Q8
IF Q4 = b (NOT citizen of Italy) or Q5 = b (Citizen of Italy NOT from birth) → GO TO Q6

Q6. In which country were you born?

WESTERN EUROPE	*EASTERN EUROPE*	*NORTH AFRICA*	*RUSSIA AND CENTRAL ASIA*	*EAST ASIA*	*SOUTHEAST ASIA*	*MIDDLE EAST*	*LATIN AMERICA*	*NORTH AMERICA AND OCEANIA*
France	Albania	Morocco	Russia	China	India	Pakistan	Peru	United States
Germany	Romania	Egypt	Central Asia	Japan	Sri Lanka	Afghanistan	Chile	Canada
Spain	Ukraine	Tunisia	Other (Please	Other (Please	Bangladesh	Syria	Brazil	Australia

[1] Recoded into: 18-24; 25-34; 35-44; 45-54; 55-64; 65-70.

			specify) OPEN	specify) OPEN				
United Kingdom	Poland	Other (Please specify) OPEN			Vietnam	Iraq	Colombia	Other (Please specify) OPEN
Netherlands	Other (Please specify) OPEN				Other (Please specify) OPEN	Other (Please specify) OPEN	Argentina	
Belgium							Other (Please specify) OPEN	
Ireland								
Other (Please specify) OPEN								

After Q6, IF Q4 = b (NOT citizen of Italy) or Q5 = b (Citizen of Italy NOT from birth)

Q7. What year did you first come to live in Italy?
DIGITE /…………………/

Q8. Which of the following terms would you use to describe the place where you live?

a) A large city (> 1 million inhabitants)
b) A municipality near a large city
c) A city with 50,000 to 1 million inhabitants
d) A small town or urban village (less than 50,000 inhabitants)
e) A rural village (less than 2,000 inhabitants)
f) A farm or house in the countryside

Q9. In which province do you live? SHOW PROVINCE[2]

[2] Recoded into region and macro-regions.

Q10. What is the highest level of education you have completed? If you completed your education abroad, choose the most equivalent option[3].

a) At most, primary school diploma
b) Lower secondary school diploma
c) Professional qualification (3 years in a vocational school)
d) High school diploma (Lyceums)
e) High school diploma (Technical or vocational institutes)
f) Advanced non-university training diploma (ITS, SSML, etc.)
g) Bachelor's degree or first-level master's degree
h) Master's degree (or equivalent) or second-level master's degree
i) Doctorate or equivalent level

Q11. What is your current marital status?

a) Legally married
b) In a legally registered civil union
c) In a legally recognized cohabitation
d) In an informal cohabitation – not legally recognized
e) Legally separated/ divorced/ dissolved civil union
f) Widowed / Civil partner deceased
g) Single (Never married)

Q12. How many children do you have (including biological, step, adopted, or foster children)?

(Dropdown menu – Including 0) from 0 to 12

Q13. Do you intend to have (another or your first) child within the next three years?

a) Yes
b) No
c) I do not know
d) Not applicable

Q14. If there were no constraints or obstacles, how many children would you want to have in total? Please indicate the number.

(Dropdown menu – Including 0)

a) Not applicable

[3] Recoded into "Low" (Lower secondary education or less [categories a, b, c]), "Medium" (Upper secondary or advanced technical education [categories (d, e, f)], "High" (University degree or higher [categories g, h, i].

Q15. Including yourself, how many people — including children — regularly live as members of your household? By household, we mean you and any family members living with you, excluding renters or housemates.

DIGITE /…./ NUMERIC, RANGE 1-12

Q16. How many people under 16 years old are part of your household? If there are none, indicate 0.[4]

(Dropdown menù – Including 0).

Q17. How many members of your household have an income from employment?[5]

DIGITE /…./ NUMERIC, RANGE

Q18. Which of the following best describes your primary work status last week? Select one option only[6]**.**

a) In paid work (or away temporarily) (employee, self-employed, working for your family business)
b) In education, (not paid for by employer) even if on vacation
c) Unemployed and actively looking for a job
d) Unemployed, wanting a job but not actively looking for a job
e) Permanently sick or disabled
f) Retired
g) In community or military service
h) Doing housework, looking after children or other persons
i) Other

IF Q18 = a, b, c, d, f → GO TO Q19, Q20
IF Q18= e, g, h, i → GO TO Q22

Q19. What is your current occupation? If you are retired or unemployed, please specify your last occupation. Select one option only.

a) Intellectual, professional, and scientific professions (e.g., doctor, teacher, engineer, artist, accountant, commercialist)
b) Directors, managerial functions in administration (e.g., banker, CEO of a large company, state councillor, trade union leader)
c) Clerical sector (e.g., secretary, office employee, service head, accountant)
d) Sales sector (e.g., sales representative, shopkeeper, store clerk, insurance agent, representative)
e) Service sector (e.g., restaurateur, police officer, waiter, janitor, hairdresser, non-commissioned military officer)
f) Skilled worker (e.g., construction foreman, mechanic, typographer, tool and die maker, electrician)
g) Semi-skilled worker (e.g., bricklayer, bus driver, worker in food processing, carpenter, metal worker, baker)

---

[4] OPEN, NUMERIC, RANGE 0-11, the number must be < than Q16. Apply controls: the number must be less than the value entered in question 16.
[5] OPEN, NUMERIC, RANGE 0-12, check, the number must be = or < than the one in Q16.
[6] Recoded into: EMPLOYED: a + g; UNEMPLOYED: c; INACTIVE: b + d + e + f + h; OTHER: i.

h) Unskilled worker (e.g., general worker, porter, unskilled labourer, cleaner)
i) Agricultural worker (e.g., farmer, agricultural worker, tractor driver, fisherman)
j) Owner or manager of an agricultural business
k) Don't know

Q20. Which of the following best describes your occupation? If you are retired or unemployed, please specify your last occupation.

a) Employee
b) Self-employed without employees
c) Self-employed with fewer than 15 employees
d) Self-employed with at least 15 employees
e) Family worker

IF Q20= a→ GO TO Q21
IF Q20= b, c, d, e → GO TO Q22

Q21. In your primary occupation, do/did you have direct responsibility for supervising the work of other employees?

a) Yes
b) No

Q22. Consider your household. What are the MAIN sources of income for your household? Select all that apply.

a) Income from salaried employment
b) Income from self-employment (excluding agriculture)
c) Income from agriculture
d) Pensions
e) Unemployment benefits/cessation allowance
f) Other social benefits or subsidies
g) Income from investments, savings, insurance, or property
h) Income from other sources

Q23. What is your household's NET MONTHLY INCOME? Consider the monthly income remaining after paying taxes and all deductions.

a) Less than 250
b) 251-500
c) 501 a 750
d) 751 a 1000
e) 1001 a 1250

f) 1251 a 1500
g) 1501 a 1750
h) 1751 a 2000
i) 2001 a 2250
j) 2251 a 2500
k) 2501 a 2750
l) 2751 a 3000
m) 3001 a 3250
n) 3251 a 3500
o) 3501 a 3750
p) 3751 a 4000
q) 4001 a 4250
r) 4251 a 4500
s) 4501 a 4750
t) 4751 a 5000
u) 5001 a 5250
v) 5251 a 5500
w) 5501 a 5750
x) 5751 a 6000
y) Over 6000

Q24. The question about your household's average net monthly income is crucial for our research. Please indicate the <u>EXACT net MONTHLY INCOME</u> of your household. Please only allow ranges that are in line with what is indicated in Q23.

DIGITE /…………………./ (Numeric)

a) I do not know

Q25. With your household's net monthly income, how difficult is it for you to make ends meet at the end of the month?

a) No difficulty
b) A little difficulty
c) Some difficulty
d) A lot of difficulty

Q26. Which of the following statements best describes your housing situation?

a) The main residence is owned by me or another member of my household
b) The main residence is rented by me or another member of my household
c) The main residence is owned by others, where my family and I live rent-free
d) Other situation

Q27. How religious do you consider yourself?

1 – Not religious at all	2	3	4	5	6	7	8	9	10 – Very religious

a) Prefer not to answer

Q28. In politics people sometimes talk of "left" and "right". Using this card, where would you place yourself on this scale, where 0 means the left and 10 means the right?

1- Left	2	3	4	5	6	7	8	9	10 - Right

a) Prefer not to answer

Q29. How much trust do you have in the following institutions? Use a scale from 1 to 10, where 1 means "No trust at all" and 10 means "Complete trust".

a) In the Italian state	1	2	3	4	5	6	7	8	9	10
b) In the legal system	1	2	3	4	5	6	7	8	9	10
c) In the police	1	2	3	4	5	6	7	8	9	10
d) In the politicians	1	2	3	4	5	6	7	8	9	10
e) In the political parties	1	2	3	4	5	6	7	8	9	10
f) In the European Union	1	2	3	4	5	6	7	8	9	10
g) In the United Nations	1	2	3	4	5	6	7	8	9	10

Q30. On a typical day, through which means do you keep yourself informed? <u>Select the most important source first, then the second and third (up to three responses)</u>

a) Online social media (e.g., WhatsApp, Facebook, TikTok, Instagram, YouTube, Twitter, Telegram)
b) Television
c) Newspapers (in print or accessed via the internet)
d) Radio and podcasts (including internet radio or access via the internet)
e) Websites (including exclusively online news sites, news aggregators, portals, or search engines)
f) Oher
g) None

Q31. Would you describe yourself as being a member of a group that is discriminated against in this country?

a) Yes
b) No
c) Prefer not to answer

IF Q31= a→ GO TO Q32
IF Q31= b, c → GO TO Q33

Q32. On what grounds is your group discriminated against? Select all that apply.

a) Ancestry or national origins
b) Religion
c) Height or weight
d) Gender
e) Skin colour
f) Sexual orientation
g) Age
h) Disability
i) Educational or work background
j) Other (Specify) ___________
k) Prefer not to answer

Q33. What is the highest level of education your father completed? If your education was completed abroad, choose the closest equivalent.

a) At most, elementary school
b) Lower secondary school or vocational qualification (3 years of vocational school)
c) High school diploma or non-university advanced vocational training (ITS, SSML, etc.)
d) University degree (Bachelor's, Master's, integrated cycle, etc.)
e) I don't know

Q34. What is the highest level of education your mother completed? If her education was completed abroad, choose the closest equivalent.

a) At most, elementary school
b) Lower secondary school or vocational qualification (3 years of vocational school)
c) High school diploma or non-university advanced vocational training (ITS, SSML, etc.)
d) University degree (Bachelor's, Master's, integrated cycle, etc.)
e) I don't know

Q35. In which country was your father born?

a) ITALY

WESTERN EUROPE	*EASTERN EUROPE*	*NORTH AFRICA*	*RUSSIA AND CENTRAL ASIA*	*EAST ASIA*	*SOUTHEAST ASIA*	*MIDDLE EAST*	*LATIN AMERICA*	*NORTH AMERICA AND OCEANIA*
France	Albania	Morocco	Russia	China	India	Pakistan	Peru	United States
Germany	Romania	Egypt	Central Asia	Japan	Sri Lanka	Afghanistan	Chile	Canada
Spain	Ukraine	Tunisia	Other (Please specify) OPEN	Other (Please specify) OPEN	Bangladesh	Syria	Brazil	Australia
United Kingdom	Poland	Other (Please specify) OPEN			Vietnam	Iraq	Colombia	Other (Please specify) OPEN
Netherlands	Other (Please specify) OPEN				Other (Please specify) OPEN	Other (Please specify) OPEN	Argentina	
Belgium							Other (Please specify) OPEN	
Ireland								
Other (Please specify) OPEN								

Q36. In which country was your mother born?

a) ITALY

WESTERN EUROPE	*EASTERN EUROPE*	*NORTH AFRICA*	*RUSSIA AND*	*EAST ASIA*	*SOUTHEAST ASIA*	*MIDDLE EAST*	*LATIN AMERICA*	*NORTH AMERIC*

			CENTRAL ASIA					*A AND OCEANIA*
France	Albania	Morocco	Russia	China	India	Pakistan	Peru	United States
Germany	Romania	Egypt	Central Asia	Japan	Sri Lanka	Afghanis tan	Chile	Canada
Spain	Ukraine	Tunisia	Other (Please specify) OPEN	Other (Please specify) OPEN	Banglad esh	Syria	Brazil	Australia
United Kingdom	Poland	Other (Please specify) OPEN			Vietnam	Iraq	Colombi a	Other (Please specify) OPEN
Netherla nds	Other (Please specify) OPEN				Other (Please specify) OPEN	Other (Please specify) OPEN	Argentin a	
Belgium							Other (Please specify) OPEN	
Ireland								
Other (Please specify) OPEN								

Q37. How much do you agree or disagree with the following statements? Please rate on a scale from 1 to 5, where 1 means "Strongly disagree" and 5 means "Strongly agree".

	Strongly disagree	Rather disagree	Neither disagree nor agree	Rather agree	Strongly agree
a) When jobs are scarce, men should have more right to a job than women.	1	2	3	4	5

b) Homosexual couples are as good parents as other couples.	1	2	3	4	5
c) Men are more competent than women in holding positions of authority.	1	2	3	4	5

Gender inequality

Q38. Thinking about the current situation in Italy, would you say that men and women are treated equally?

a) Yes, men and women are treated equally
b) No, women are disadvantaged compared to men
c) No, men are disadvantaged compared to women

Q39. In your opinion, the inequalities between men and women in Italy currently are:

a) A serious problem
b) A somewhat serious problem
c) A minor problem
d) Not a problem at all

Q40. In your opinion, compared to ten years ago, the inequalities between men and women in Italy have:

a) Increased a lot
b) Increased somewhat
c) Stayed the same
d) Decreased somewhat
e) Decreased a lot

Q41. Comparing your region with Italy as a whole, would you say that the level of inequality between men and women in your region is lower, higher or about the same?

a) Lower
b) Higher
c) About the same

Q42. Public and private institutions are implementing a series of policies and measures to promote equal opportunities in the workplace and education, and to protect discriminated groups (positive actions). In principle, how much do you approve or disapprove of these actions?

a) Strongly disapprove
b) Disapprove
c) Neither approve nor disapprove
d) Approve
e) Strongly approve

f) Prefer not to answer

Q43. How much do you agree or disagree with supporting the following types of positive actions? By "quota", we mean a specific percentage of positions reserved for certain groups of people.

	Strongly disagree	Rather disagree	Neither disagree nor agree	Rather agree	Strongly agree
a) Representative quotas of women in politics					
b) Quotas of women on boards of directors					
c) Representative quotas of LGBTQIA+ people in politics.					
d) Representative quotas of LGBTQIA+ people on the Boards of Directors					

Income inequality

Q44. These five diagrams show different types of society. Please read the descriptions and look at the diagrams and decide which you think best describes Italy. First, what type of society is Italy today – which diagram comes closest?

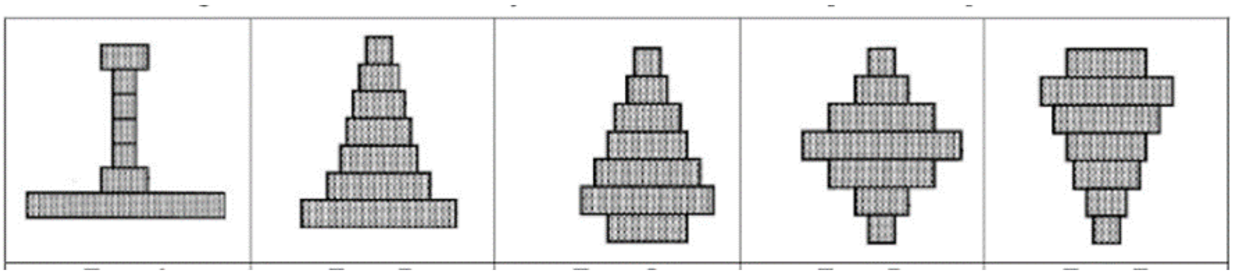

Type A	Type B	Type C	Type D	Type E
A small elite at the top, very few people in the middle, and the vast majority at the bottom.	A society shaped like a pyramid with a small elite at the top, more people in the middle, and the majority at the bottom.	A pyramid, except that only a few people are at the bottom.	A society where the majority of people are in the middle.	Many people near the top and only a few near the bottom.

a) Type A
b) Type B
c) Type C
d) Type D
e) Type E

Q45. What do you think Italy ought to be like – which would you prefer??

a) Type A
b) Type B
c) Type C
d) Type D
e) Type E

Q46. Thinking about income inequality in Italy from 2000 to today, do you believe inequality has decreased or increased?

a) Decreased a lot

b) Decreased
c) Stayed the same
d) Increased
e) Increased a lot

Q47. Looking to the future, do you believe income inequality in Italy will decrease or increase in the next 20 years?

a) Decrease a lot
b) Decrease
c) Stay the same
d) Increase
e) Increase a lot

Q48. Comparing your region with Italy as a whole, would you say that the level of income inequality in your region is lower, higher or about the same?

a) Lower
b) Higher
c) About the same

Q49. In general, to what extent do you agree or disagree with the following statements? Select one box for each row from 1 to 10, where 1 means "Strongly disagree" and 10 means "Strongly agree”.

a) Income differences are too large.	1	2	3	4	5	6	7	8	9	10
b) The government should reduce the income gap between the rich and the poor.	1	2	3	4	5	6	7	8	9	10
d) Redistributive policies, when adopted by governments, reduce poverty.	1	2	3	4	5	6	7	8	9	10
e) The free market reduces poverty	1	2	3	4	5	6	7	8	9	10
f) All countries should open their borders and welcome asylum seekers	1	2	3	4	5	6	7	8	9	10

g) All countries should open their borders, but not offer full rights as citizens to migrants	1	2	3	4	5	6	7	8	9	10

Q50. If you think of the future of the younger generation in Italy, do you think this generation, compared to their parents in terms of social mobility, will have..:

a) Significantly fewer opportunities
b) Somewhat fewer opportunities
c) The same opportunities
d) Somewhat more opportunities
e) Significantly more opportunities

Q51. On a scale from 1 to 5, where 1 means "Not important at all" and 5 means "Very important," how important do you think the following conditions are for success in life in Italy?

	Not at all important	Little important	Neither irrelevant nor important	Enough important	Very important
a) Coming from a wealthy family	1	2	3	4	5
b) Having a good level of education	1	2	3	4	5
c) Working hard	1	2	3	4	5
d) Having talent	1	2	3	4	5
e) Knowing the right people	1	2	3	4	5
f) Having political support	1	2	3	4	5
g) Paying bribes or engaging in corruption	1	2	3	4	5

Q52. Currently, there are about 59 million people in Italy. Of these 59 million people, how many do you think have a lower net monthly income per capita than yours? Please use the slider below.

[CURSOR]

0*******30*******59 million people

0*****************100%

0	6 mln	12 mln	18 mln	24 mln	30 mln	36 mln	42 mln	48 mln	54 mln	60 mln
COLORED SLIDER										
0%	10%	20%	30%	40%	50%	60%	70%	80%	90%	100%

Migration and ethnic inequality

Q53. Think about the foreign population residing in Italy. In your opinion, what are the three main geographical areas they come from? <u>Please indicate the first, second, and third areas</u>.

a) Western Europe (Germany, Spain, United Kingdom…)
b) Eastern Europe (Albania, Romania, Ukraine, Poland…)
c) North Africa (Morocco, Egypt, Tunisia…)
d) Sub-Saharan Africa (Senegal, Ghana, Nigeria, Eritrea, Somalia…)
e) Russia and Central Asia (Kazakhstan, Uzbekistan,…)
f) East Asia (China, Japan,…)
g) Southeast Asia (India, Sri Lanka, Bangladesh, Vietnam…)
h) Middle East (Pakistan, Afghanistan, Syria, Iraq…)
i) Latin America (Peru, Chile, Brazil, Colombia, Argentina…)
j) North America and Oceania (United States, Canada, Australia…)
k) Other (Please specify)

Q54. Think about the majority of foreigners residing in Italy and use the slider to evaluate them based on the following traits.

Not sincere ------------ Sincere
Rude ------------ Polite
Hostile ------------ Friendly
Weak ------------ Strong
Incompetent ------------ Competent
Lazy ------------ Hardworking
Insecure ------------ Sure
Without ambition ------------ Competitive
Submissive ------------ Independent
Dangerous ------------ Harmless
Cold ------------ Warm

Q55. Think about the majority of Italians and use the slider to evaluate them based on the following traits.

Not sincere ------------ Sincere
Rude ------------ Polite
Hostile ------------ Friendly
Weak ------------ Strong
Incompetent ------------ Competent
Lazy ------------ Hardworking
Insecure ------------ Sure
Without ambition ------------ Competitive

Submissive ------------ Independent
Dangerous ------------ Harmless
Cold ------------ Warm

Q56. People migrate for different reasons. In your opinion, why did most foreigners residing in Italy come to Italy? <u>Select the most important reason first (maximum two answers).</u>

a) To escape wars, armed conflicts, and human rights violations
b) To work in paid jobs / find better job opportunities
c) To reunite with family members who emigrated earlier
d) For educational purposes
e) To seek better living conditions due to environmental conditions (droughts, floods, catastrophes, etc.) where they live
f) Other (please specify) **open, anchor**

Q57. Think about the majority of foreigners residing in Italy. On a scale from 1 to 5, where 1 means "Never" and 5 means "Always," how often, in your opinion, do they experience discrimination because of their ethnicity?

	Never	Almost never	Some time	Often	Always
a) At work	1	2	3	4	5
b) When applying for a job	1	2	3	4	5
c) When renting or buying real estate	1	2	3	4	5
d) By the police	1	2	3	4	5
e) On the street or in public	1	2	3	4	5
f) In personal interactions	1	2	3	4	5

Q58. In your opinion, international migration worldwide should...

a) Increase a lot
b) Increase
c) Stay the same
d) Decrease
e) Decrease a lot

Global inequality

Q59. On a scale from 1 to 10, where 1 means "Strongly disagree" and 10 means "Strongly agree," to what extent do you agree or disagree with the following statements.

a) Present economic differences between rich and poor countries are too large.	1	2	3	4	5	6	7	8	9	10
b) People in wealthy countries should make an additional tax contribution to help people in poor countries.	1	2	3	4	5	6	7	8	9	10
c) People from poor countries should be allowed to work in wealthy countries.	1	2	3	4	5	6	7	8	9	10

Q60. Comparing Italy with the rest of the European Union, would you say that income inequalities are higher, lower or about the same?

a) Higher
b) Lower
c) About the same

Q61. Currently, there are about 8 billion people in the world. Of these 8 billion people, how many do you think have a lower net monthly income per capita than yours? Please use the slider below.

[CURSOR]
0*******4*******8 billion people
0—-10%—-20%—--30%—---40%—-50%—-60%----70%—-80%—-90%—--100%

0	0,8 mld	1,6 mld	2,4 mld	3,2 mld	4 mld	4,8 mld	5,6 mld	6,4 mld	7,2 mld	8 mld
COLORED SLIDER										
0%	10%	20%	30%	40%	50%	60%	70%	80%	90%	100%

Q62. Imagine it is possible to redistribute income globally in a way similar to how a state can redistribute income within a country. On a scale from 1 to 10, where 1 means "No redistribution" and 10 means "Complete redistribution," how much income redistribution would you want between the citizens of the world?

No redistribution means that the global distribution of income is not influenced. Complete redistribution means that everyone in the world earns the same income after redistribution.

1 – No redistribution	2	3	4	5	6	7	8	9	10 – Complete redistribution

MODULE – Income and social status

Poverty and Redistribution Preferences: This experiment evaluates how individuals' attitudes toward redistribution are affected by exposure to information about relative poverty levels and their position in income distribution with reference to poverty line. It explores whether personal experience and increased awareness of their own self-interest in redistribution shape their support for redistributive policies.

For further information, see: https://osf.io/84njk.

MODULE – Gender gap and fairness

Gender Gap and Fairness: The gender pay gap experiment is a factorial survey (vignette) study that explores perceptions of fair wages and candidate suitability based on various characteristics. This study investigates how different factors (e.g., age, ethnicity, parental status, field of study, occupation, and hobbies) interact with the gender of the candidate to influence respondents' evaluations of ideal salary and job suitability. Through this experimental design, we aim to disentangle the underlying reasons for the gender pay gap. For further information, see: https://osf.io/6q4f8.

MODULE – Gender-based violence

Perceptions and Normalization of Gender-Based Violence: The normalization of gender-based violence is examined through a factorial survey (vignette) experiment that assesses the acceptance of various situations, which vary randomly across dimensions, and the willingness to intervene. his study explores both societal and individual acceptance of ambiguous or more subtle forms of gender-based violence, as well as identifies the situational factors that increase or decrease the likelihood of bystander intervention. For further information, see: https://osf.io/zbgh6.

After the vignettes

Questions from GG4 to GG6 are out of the loop and must be asked only once after the 4 scenarios. When asking from GG4 to GG6 no need to display the scenario above the questions

GG4. In the past year, have you or someone you know been subjected to any of the following situations?

	Yes	No	I do not know	I don't want to answer
a) Domestic violence or abuse.				
b) Economic violence, such as denying access to money or banning attendance at school or work.				
c) Psychological violence by his/her partner, such as acts, words, threats and intimidation used as a tool of coercion and oppression				

GG5. In the past year, have you or someone you know been subjected to the following situations while carrying out your work?

	Yes	No	I do not know	I don't want to answer
a) Verbal abuse				
b) Unwanted sexual attention				
c) Threats				
d) Humiliating behaviour				
e) Physical violence				
f) Sexual harassment				
g) Bullying / harassment				

GG6. On a scale from 1 to 5, where 1 means "Very rare" and 5 means "Very common," how common or rare do you think these types of violence are in Italy? Single per item, rotate items

	Very rare	Rare	Neither rare nor common	Common	Very common
a) Violence against women due to their gender.					
b) Violence against men due to their gender.					
c) Violence against the LGTBQIA+ community due to their sexual orientation and/or gender identity.					

MODULE – Global challenges

This module includes two different experiments:

1. *Perceptions of Asylum Seekers and Refugees:* Attitudes toward international migration are studied through an image-based vignette experiment that explores how respondents' evaluations change based on characteristics, which vary randomly across dimensions, depicted in photographs. This study investigates how these features influence respondents' assessments of perceived vulnerability, asylum requests, and support for government interventions such as financial assistance, housing, and Italian language courses, which can facilitate the integration of asylum seekers. For further information, see: https://osf.io/kuf4e.

2. *Climate Policy Preferences and Perception of Climate Fairness in Italy:* The climate policy preference experiment investigates public support for climate mitigation policies and assesses their fairness through a conjoint experiment. It explores how respondents' ideology (e.g., egalitarian orientations), trust in institutions, and perceived proximity to climate change impacts (based on psychological distance theory) shape preferences for policy packages and their perceived fairness. For further information, see: https://osf.io/b4fgx.

Questions before the experiment *Climate Policy Preferences and Perception of Climate Fairness in Italy:*

F1. In 2009, scientists proposed the idea of "planetary boundaries" to help us understand and stay within safe limits for the health of the Earth. Using the following indicator, please evaluate the situation for each of the following subsystems in 2023.

F1_1. CO2 – quantity and concentration of CO2 (carbon dioxide)

SLIDER: Far below the limit --- Below the limit ----- X (middle point) ----- Above the limit ---- Far above the limit

F1_2. Fresh water – consumption of fresh water by humans

SLIDER: Far below the limit --- Below the limit ----- X (middle point) ----- Above the limit ---- Far above the limit

F1_3. Land use – percentage of global land cover converted to arable land

SLIDER: Far below the limit --- Below the limit ----- X (middle point) ----- Above the limit ---- Far above the limit

F2. How concerned are you about climate change?

a) Not at all worried
b) Not very worried
c) Rather worried
d) Very worried
e) Extremely concerned

F3. Thinking about the causes of climate change, which of the following descriptions best reflects your opinion?

a) Climate change does not exist
b) ...mainly caused by natural processes
c) ...partly caused by natural processes and partly by human activities
d) ...mainly caused by human activities

MODULE – Migration

Migrant Children's Share and Threat Perceptions: This experiment investigates whether presenting objective data on the proportion of children with migrant backgrounds in Italy affects public perceptions of migration and attitudes toward immigrants. The experiment examines whether factual data can alter perceptions of threat or influence attitudes towards migrants.

For further information, see: https://osf.io/bfz84/.
